# Nitric oxide is cytoprotective to breast cancer spheroids vulnerable to estrogen-induced apoptosis

**DOI:** 10.18632/oncotarget.21610

**Published:** 2017-10-07

**Authors:** Yana Shafran, Naomi Zurgil, Orit Ravid-Hermesh, Maria Sobolev, Elena Afrimzon, Yaron Hakuk, Asher Shainberg, Mordechai Deutsch

**Affiliations:** ^1^ The Biophysical Interdisciplinary Jerome Schottenstein Center for the Research and Technology of the Cellome, Physics Department, Bar Ilan University, Ramat Gan 52900, Israel; ^2^ The Mina and Everard Goodman Faculty of Life Sciences, Bar Ilan University, Ramat Gan 52900, Israel

**Keywords:** nitric oxide, breast cancer spheroids, estrogen-induced apoptosis, estrogen receptor-positive, live-cell imaging

## Abstract

Estrogen-induced apoptosis has become a successful treatment for postmenopausal metastatic, estrogen receptor-positive breast cancer. Nitric oxide involvement in the response to this endocrine treatment and its influence upon estrogen receptor-positive breast cancer progression is still unclear.

Nitric oxide impact on the MCF7 breast cancer line, before and after estrogen-induced apoptosis, was investigated in 3D culture systems using unique live-cell imaging methodologies.

Spheroids were established from MCF7 cells vulnerable to estrogen-induced apoptosis, before and after exposure to estrogen.

Spheroids derived from estrogen-treated cells exhibited extensive apoptosis levels with downregulation of estrogen receptor expression, low proliferation rate and reduced metabolic activity, unlike spheroids derived from non-treated cells. In addition to basic phenotypic differences, these two cell cluster types are diverse in their reactions to exogenous nitric oxide.

A dual effect of nitric oxide was observed in the breast cancer phenotype sensitive to estrogen-induced apoptosis. Nitric oxide, at the nanomolar level, induced cell proliferation, high metabolic activity, downregulation of estrogen receptor and enhanced collective invasion, contributing to a more aggressive phenotype. Following hormone supplementation, breast cancer 3D clusters were rescued from estrogen-induced apoptosis by these low nitric oxide-donor concentrations, since nitric oxide attenuates cell death levels, upregulates survivin expression and increases metabolic activity.

Higher nitric oxide concentrations (100nM) inhibited cell growth, metabolism and promoted apoptosis. These results suggest that nitric oxide, in nanomolar concentrations, may inhibit estrogen-induced apoptosis, playing a major role in hormonal therapy. Inhibiting nitric oxide activity may benefit breast cancer patients and ultimately reduce tumor recurrence.

## INTRODUCTION

Breast cancer (BC) occurs in both women and men, and its incidence increases with age. BC is the most frequently diagnosed cancer among women, and the leading cause of cancer-related death worldwide [1].

Estrogen receptor (ER), detected in approximately 70 to 80% of primary BCs, regulates endocrine-dependent growth in these tumors. Expression of ER often mediates sensitivity of tumor cells to hormonal treatment. Hormonal manipulation is achieved either at a cellular level by using anti-estrogens, such as tamoxifen, to compete for ER in the breast tumor, or systemically, by lowering estrogen levels in premenopausal women by the use of luteinizing hormone-releasing hormone agonists and in postmenopausal women by aromatase inhibitors that block estrogen biosynthesis in non-ovarian tissues. These endocrine therapies are very effective as treatment for ER-positive BC, and for patients with early-stage disease, hormonal therapy given for five years after primary surgery markedly delays local and distant relapse and prolongs overall survival [2, 3]. Despite major therapeutic success, the strategies of long term anti-hormone therapies eventually lead to a significant proportion of anti-hormone resistant or stimulated tumor growth and failure of therapy is observed in 20-25% of patients who exhibit intrinsic or acquired resistance [4, 5]. These patients ultimately demonstrate further disease progression that may lead to outgrowth of metastases in distant organs and cancer-related death [6]. Much progress has been made in understanding the molecular biology associated with secondary endocrine resistance.

Interestingly, it was discovered that the cell populations which emerge and grow after long-term anti-hormone treatment are vulnerable to the cytotoxicity of estrogen-induced apoptosis. Hence, BC cell growth and BC cell apoptosis are both regulated via the ER. As a result, estrogen-induced apoptosis becomes a successful treatment for postmenopausal metastatic, ER-positive BC women [7-9]. The mechanism by which estrogens are able to induce apoptosis was suggested to be selection of BC populations that are resistant to long-term estrogen deprivation, and are vulnerable to physiologic estrogen that triggers apoptosis and tumor regression [7, 10]. Another mechanism, which is proposed by Suba in [11], is compensatory restoration of ER-signaling by estrogen, which prompts apoptotic death of tumor cells. It has been implied that estrogen withdrawal or ER blockade, induced acquired estrogen hypersensitivity which is attributed to ER overexpression and high 17β-estradiol (E2) synthesis, even after long-term antiestrogen therapy, resulting in apoptotic death of tumor cells [11, 12].

In order to amplify and enhance hormone-induced apoptosis, targeted strategies to inhibit cell survival pathways are now required [13]. This calls for specially designed, advanced cellular models and tailored assays which more accurately reproduce the *in vivo* complexity of BC cells and tumor microenvironment.

Cellular models of BC are being utilized in the laboratory to mimic the clinical status of hormone-resistant breast tumor, and function as tools to discover new therapeutic strategies. Several MCF7 BC lines were generated, based on their sensitivity to estrogen-induced apoptosis, either via long-term deprivation [14] or by induction of antiestrogen (tamoxifen) resistance [15]. In spite of some differences between the various lines, all showed apoptosis induction following exposure to low physiologic concentrations of estrogen, increased ER levels, and marked translocation of ER from the nucleus into the cytoplasm [16].

The action of E2 by ER is known to elicit nitric oxide (NO) signaling via activation of NO synthase (NOS) in many tissues. NOS is often elevated in breast tumors that lack expression of the ER, and has been proved to be a key driver of signaling pathways in ER-negative BC, which promotes tumor growth, metastasis and drug resistance [17].

However, the involvement of NO in the response to endocrine treatment in general, and especially to estrogen-induced apoptosis, as well as its contribution to ER-positive BC aggressiveness is still unclear.

Utilizing advanced cellular models, this study aims to investigate the role of NO in the response to this endocrine treatment, (e.g. estrogen-induced apoptosis) of ER-positive BC cells, in order to better understand, and ultimately reduce, tumor recurrence.

BC spheroids were established from MCF7 cells vulnerable to estrogen-induced apoptosis, before and after exposure to the hormone, and their response to exogenous NO was examined via live-cell imaging tools.

## RESULTS

### MCF7 cells vulnerable to estrogen-induced apoptosis before and after exposure to E2

**Figure 1:**
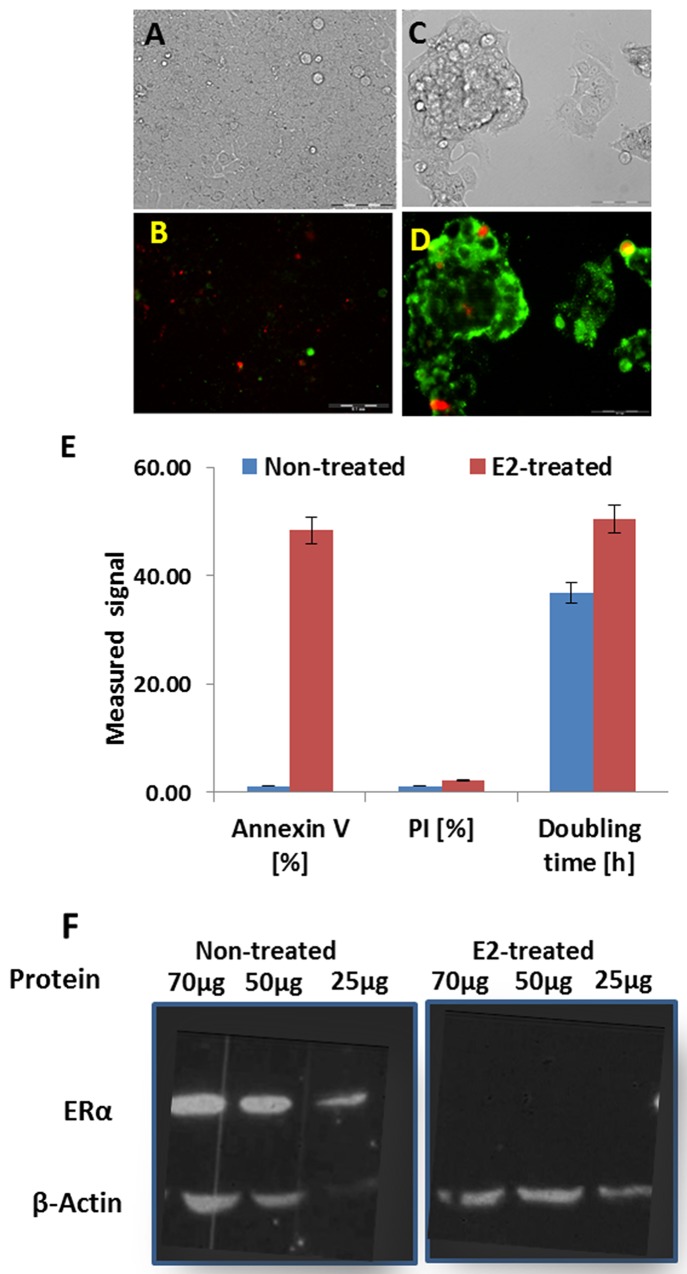
Bright field **(A,B)** and corresponding fluorescence images **(C,D)** of MCF7 cells vulnerable to E2-induced apoptosis before (A,C) and two weeks after hormonal treatment (B,D). Cells were double stained with Annexin V-FITC (green signal) and PI (red signal). **(E)** Level of apoptosis and doubling time in MCF7 cells before and after exposure to E2. **(F)** Relative expression levels of ER in non-treated (left) and E2-treated (right) MCF7 cells.

Bright field and fluorescence images of MCF7 cells grown in 2D culture, prior to and following exposure to E2 are depicted in Figure 1. Before hormonal treatment, MCF7 cells proliferate and develop homogeneous monolayer (Figure 1A). The doubling time of this phenotype is about 35.5h, and the percent of Annexin V and propidium iodide (PI)-positive cells was negligible. (1.2±0.13% and 1.1±0.1% for apoptosis and cell death signals, respectively) (Figure 1B). Upon addition of 10nM E2 to culture medium, in the course of two weeks the MCF7 BC cells undergo a slow morphological shift. Following 5 passages in the presence of medium supplemented with E2, a dramatic cell detachment is evident, the cells grow in multilayer structures (Figure 1C) and extensive apoptosis is apparent (Figure 1D). The average area stained for Annexin V reaches 48.5±7.7% and the doubling time of the cells is 50.5h, which is slower in comparison to that of non-treated cells. Concomitantly, cell death levels increased, but remained low (percent of PI stained area was 2.2±1.4%). Hence, hormonal treatment induced high levels of apoptosis and the cells were proliferating at a lower rate (Figure 1E). Upon additional culturing of the cells in the presence of E2, almost all the cells become detached from the culture plate and a remarkable decrease in cell number (up to 90%) is observed (data not shown).

The differences between the two cell phenotypes accompanied substantial variation in ERα status. Two weeks after E2-induced apoptosis, downregulation of ERα expression levels was evident, and the protein was no longer detectable by western blotting analysis (Figure 1F).

This data is in agreement with previous results by Pink et al [18], which presented a model of ER regulation in MCF7 line that involves upregulation of ER mRNA and protein in an estrogen-depleted environment, but ER is downregulated in the presence of estrogen.

### Generation and growth of spheroids from MCF7 cells before and after exposure to E2

**Figure 2:**
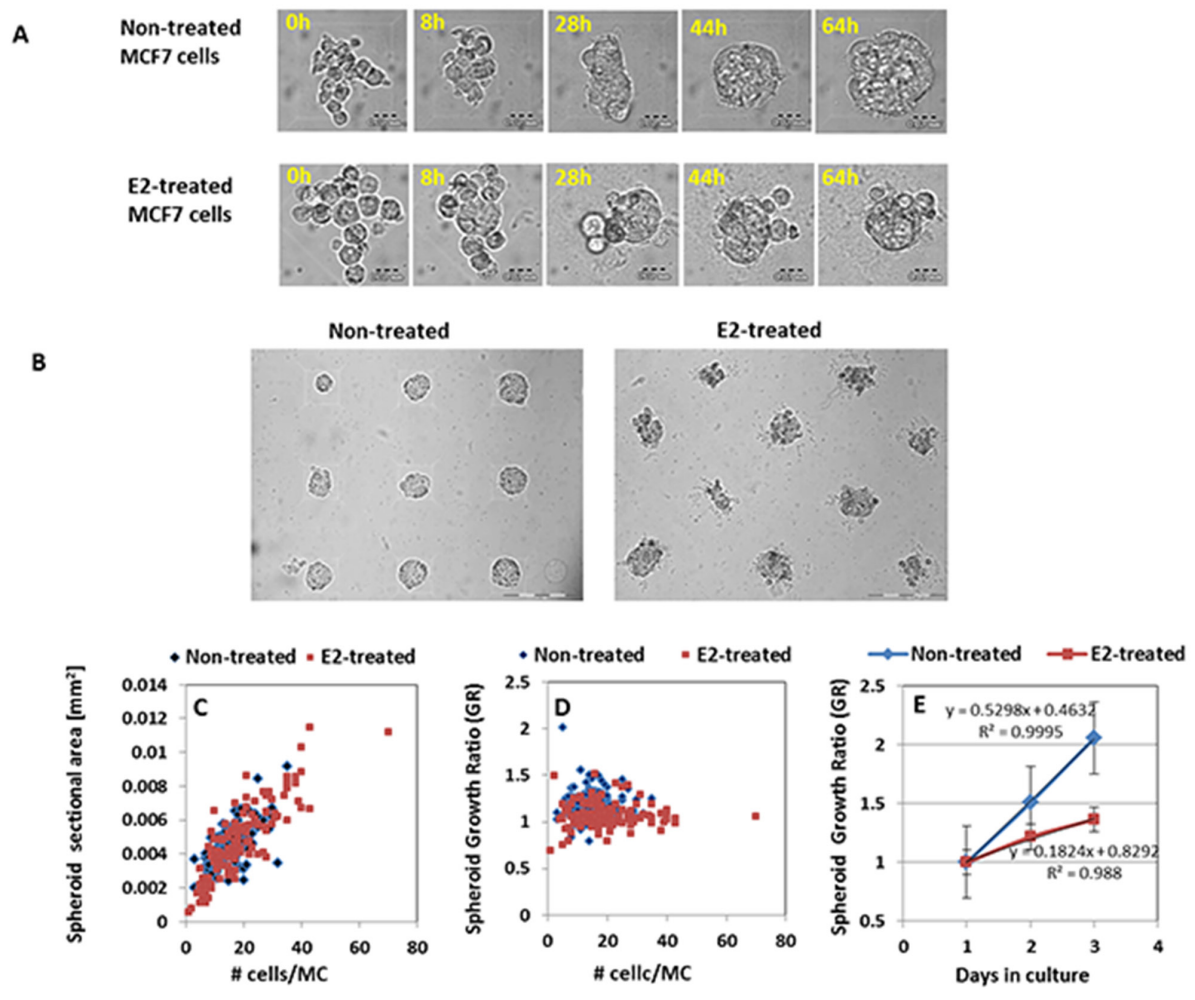
Generation and culturing of 3D multicellular MCF7 BC spheroids within HMC **(A)** kinetics of spheroid formation process from non-treated MCF7 cells (upper panel) and E2-treated (lower panel) cells. Cells were seeded within representative MCs in HMC and allowed to generate multicellular structures. Scale bar = 20μm. **(B)** Bright field images of representative 2-day BC spheroids generated within HMC array from non-treated and E2-treated MCF7 cells. Scale bar = 200μm. **(C)** Correlation between number of cells per microchamber and sectional area of spheroids at day 2. **(D)** Correlation between number of cells per microchamber and the growth rate of the corresponding spheroids. Each dot represents an individual spheroid. **(E)** Growth curves of MCF7 spheroid phenotypes within HMC array. Results represent mean ± SD (N=150).

BC spheroids were generated from MCF7 cells vulnerable to estrogen-induced apoptosis within hydrogel microchamber (HMC) arrays as described [19]. Individual MCF7 cells before and two weeks after hormonal treatment were seeded within HMC array and allowed to create cell clusters during 48h. Spontaneous creation of 3D multicellular spheroids within hydrogel array is shown in Supplementary Videos 1 and 2 (see Supplementary Materials) and Figure 2A.

During the first day after seeding, individual cells aggregate and form multicellular clusters, which continue to grow in the following days, each in an individual microchamber (MC). Both spheroid phenotypes display compact multicellular structures filled with cells, without cavities or hollows. Calculation of the number of cells comprising each spheroid showed that the increase in spheroid sectional area is associated with a parallel elevation in cell number, and hence correlates with the proliferation rate of the 3D structures (see Supplementary Material 1).

After cell seeding, due to the non-adherent nature of the hydrogel, cells retain their spherical/globular structure and their dimensions can be estimated from the bright field images (see Methods and Supplementary Material 1). At t=0, the average cell size of hormone-treated cells was significantly larger than that of MCF7 non-treated cells (Figure 2A). The corresponding average cell volumes were 1.69±0.81 pL and 2.79±1.37 pL with p<0.0001, for BC cells prior to and following exposure to E2, respectively. Representative 2-day BC spheroids spontaneously generated within HMC array from non-treated and E2-treated MCF7 cells are shown in Figure 2B. The correlation between number of cells per MC and size of the subsequently formed spheroids is shown in Figure 2C. High correlation was found between the number of cells per microchamber and the sectional area of the corresponding spheroids at day 2 for both spheroid populations (Pearson correlation=0.69 and 0.84 for spheroids derived from MCF7 cells before and after E2 treatment, respectively). Moreover, for both 3D phenotypes, the relative growth ratio (the ratio between spheroid sectional areas at two time points) of individual spheroids was not dependent on the initial number of cells per single MC (Figure 2D). Under the experimental conditions described herein (mean cell number per MC was 15±5.2), the average sectional area of 48h spheroids derived from MCF7 cells before E2 exposure was 4900±2450μm^2^, similar to the size of spheroids derived from hormone-treated cells (4880 ±1950μm^2^). However, since individual cells within two spheroid phenotypes retained their size differences, the MCF7 cell clusters which originated from hormone-treated cells contained about 30% lower number of cells in comparison to spheroids of the same size initiated from cells before hormonal treatment. Average quantities of cells were 94±32 cells vs 154±54 cells for 2-day spheroids derived from hormone-treated and non-treated cells, respectively.

Once established, the growth rate of spheroids from non-treated cells was significantly higher than that of MCF7 cell clusters originated from E2-treated cells (Figure 2E). As seen, during the second day in culture, the mean size of non-treated spheroid populations increased by 23%, while E2-treated clusters increased by only 13%. Overall, the rate of size increase during 3 days in culture was almost three times higher in cancer spheroids grown in the absence of E2 than in clusters cultured in E2-supplemented medium. The corresponding doubling time of the cluster cells were 31h and 23h for hormone-treated and non-treated 3D structures, respectively. Hence, the E2-treated spheroid phenotype displays bigger cell size and proliferates at a slower rate.

In addition, the multicellular structures derived from E2-treated and non-treated cells were morphologically different. Two-day MCF7 spheroids exhibited round and smooth shapes, while spheroids established from E2-treated cells were less spherical and had rough texture. The average sphericity factor (which describes the *roundness* of a particle by using central moments) of MCF7 non-treated spheroids was significantly higher than that of spheroids originated from E2-treated cells (0.81±0.11 and 0.62±0.16, p<0.005, respectively). Concurrently, the average parameters that define spheroid smoothness or granularity (entropy, Standard Deviation (SD) and range of gray values) were all significantly higher (1.18±0.6, 150.9±120.5, 578.4±451, respectively) for the E2-treated spheroids than for the non-treated (0.7±0.4, 67.1±37.7, 259.8±146.2, respectively) (p< 0.005 for all parameters).

### Effect of NO on spheroid growth

For analysis of the effect of exogenous NO on MCF7 BC clusters, 2-day spheroids were exposed to NO releasing compound at different concentrations for 24h. The same cell clusters were monitored before, during and after NO exposure and the ratio between spheroid sectional area before and after treatment, which reflected the change in spheroid size during treatment, was calculated and expressed as growth ratio (GR).

**Figure 3:**
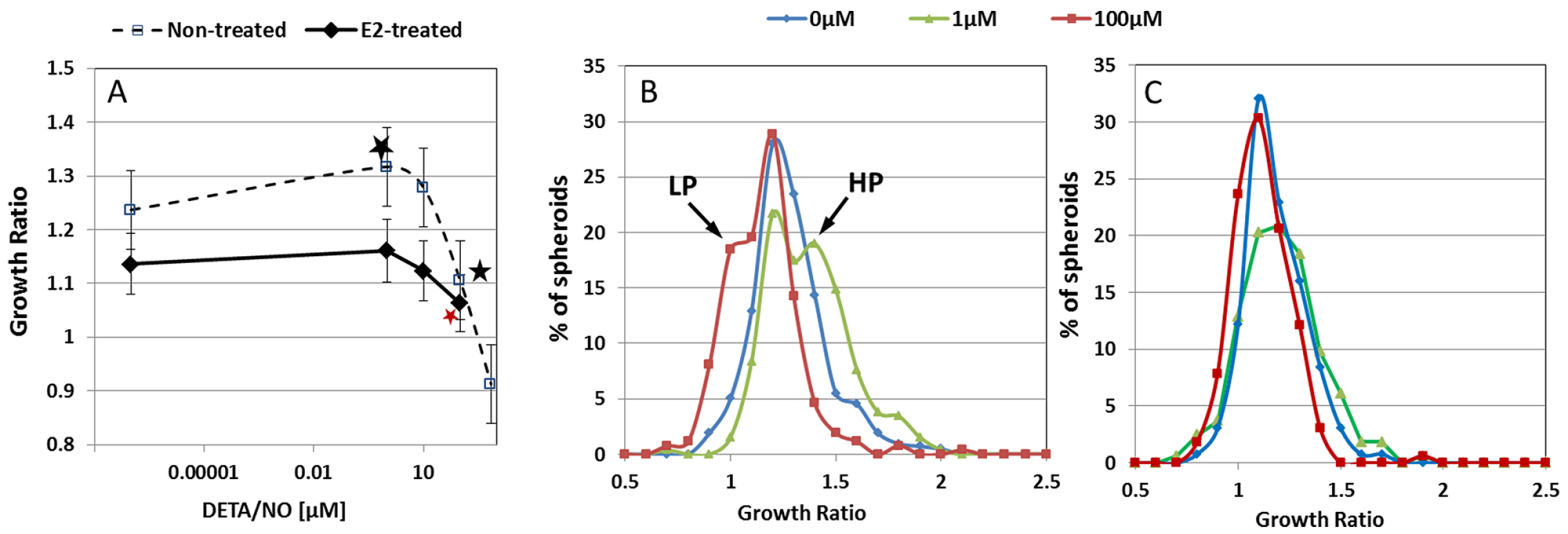
The effect of NO on spheroid growth **(A)** Dose response curve of the impact of DETA/NO on spheroid growth. Asterisks represent statistically significant differences from 0μM NO donor. Distribution histograms of MCF7 spheroid GR before **(B)** and after E2 treatment **(C)** upon incubation with DETA/NO at 1μM and 100μM. Arrows represent high (HP) and low (LP) proliferating subgroups, correspondingly.

The dose response curve for Diethyle-netriamineNONOate (DETA/NO) effect on spheroid growth is depicted in Figure 3. Without hormonal treatment, significant alterations in growth rates were evident when spheroids were incubated for 24h with the NO mimetic, DETA/NO, at both low and high concentrations (Figure 3A).

A bimodal response is evident; low doses of DETA/NO (up to 1μM) induced a significant increase in growth rate, while in the presence of higher concentrations of NO donor, the GR considerably decreased. The average increase in spheroid area for control spheroid population was 23.7±0.9 % and for 1μM DETA/NO-treated spheroids 27.8±0.9 %, while an 8.68±0.1% decrease in spheroid size was evident upon treatment with 100μM DETA/NO (p <0.0005 in both cases). Conversely, no significant change in the size of MCF7 spheroids derived from E2-treated cells was apparent upon treatment with low concentration of NO donor and a small, but significant reduction in the average dimensions was observed following exposure to higher concentrations of DETA/NO (Figure 3A).

Distribution histograms of the GR values measured in individual spheroids generated from E2-treated and non-treated MCF7 cells are shown in Figure 3B,3C. Upon exposure of non-treated 3D structures to 1μM DETA/NO, a subgroup of spheroids which showed higher relative growth rates was exposed, while with exposure of the same non-treated 3D structures to 100μM DETA/NO, a slow-growing subgroup presented. When choosing GR values of 1.43 and 1.03 as the higher and lower cutoff levels respectively (mean ± SD of the control cell population, n=400 spheroids), 31% of the MCF7 spheroids incubated in the presence of exogenous NO at 1nM concentration, displayed a significant increase in size and were labeled “high-proliferating (HP) group” (Figure 3B). Additionally, a decrease in spheroid size, induced by higher NO levels was evident in a group that comprised about 35% of the spheroid population, and labeled “low-proliferating (LP) group” (Figure 3B). The average GR value of the HP subpopulation was 1.59±0.18, while that of the LP subpopulation was 0.97±0.09. The parallel changes in mean cell number per spheroid in this phenotype were a twofold increase in HP subgroup (from 116±33.1 to 232±45.1 cells) and a 7% decrease in cell number for the LP group (from 122±29.3 to 114±25 cells), indicating an augmented propagation for the HP clusters and a cessation in cell proliferation with shrinkage of the LP spheroid subpopulation.

It should be noted that no correlation was found between the initial spheroid size and the rate of its growth in the presence or in the absence of DETA/NO. Pearson correlation of individual spheroid size and GR was: 0.042, -0.24363 and -0.19028 for control, 100 and 1μM DETA/NO, respectively. However, the HP group which appeared following 24h incubation in the presence of low NO concentration, is characterized by a significantly small sectional area at t=0 (about 25% less), which may indicate that the small dimension of these spheroids facilitates rapid diffusion and the action of NO gas molecules.

Conversely, in MCF7 clusters generated from cells exposed to E2, no specific spheroid classes were manifest upon treatment either with low or with higher DETA/NO concentrations (Figure 3C).

### Effect of NO on apoptosis level and metabolic state

**Figure 4:**
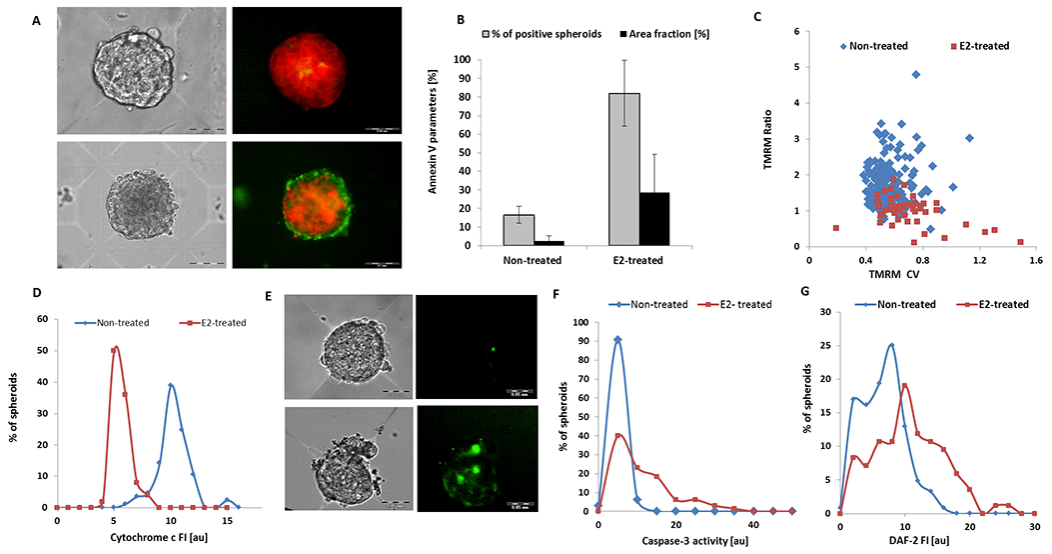
Metabolic activity and apoptosis of MCF7 spheroids in HMC array **(A)** Representative 3-day spheroids derived from non-treated (upper panel) and E2-treated cells (lower panel), stained for TMRM and Annexin V. Bright field (left panel) and overlapped fluorescent image of TMRM and Annexin V (right panel). Bars represent 100μm and 50μm for upper and lower panels, respectively. Annexin V **(B)** and TMRM fluorescence parameters **(C)** measured in populations of 3D structures generated from E2-treated and non-treated cells. **(D)** Distribution histograms of cytochrome C level reflected by anti-cytochrome C FI, measured in individual hormone-deprived and hormone-treated MCF7 spheroid populations. Note that when cytochrome c is translocating from the mitochondria to the cytosol during apoptosis process, the mean FI of cytochrome c signal is decreased. **(E)** Representative 3-day spheroids derived from non-treated (upper panel) and E2-treated (lower panel) cells, vitally stained for caspase-3 activity using NucView™ 488. Bright field (left panel) and fluorescent image of enzyme activity (right panel). Bars represent 50μm. **(F)** Distribution histograms of caspase-3 activity measured in individual MCF7 3D structures generated from non-treated and hormone-treated cells. **(G)** Distribution histograms of relative intracellular NO levels as reflected by DAF-2 FI measured in individual hormone-deprived and hormone-treated BC spheroid populations.

For the assessment of metabolic state and apoptosis level, both types of spheroids were double-stained with tetramethylrhodamine methyl ester perchlorate (TMRM) and Annexin V respectively. Representative 3-day MCF7 spheroids derived from non-treated and E2-treated cells following vital staining with TMRM and Annexin V are shown in Figure 4A. As seen, in the absence of E2, Annexin V staining is almost negligible, while homogeneous high intensity TMRM staining is evident. However, 3D structures exhibited high signals of Annexin V probe and lower scattered TMRM signals following hormonal exposure.

As expected, Annexin V results showed a high level of early apoptosis in MCF7 spheroids exposed to E2. The proportion of Annexin V-positive spheroids and averaged area fraction of the fluorescent signal are significantly higher in this spheroid population (Figure 4B).

TMRM, which accumulates within mitochondria in inverse proportion to Δψ_m_, is utilized for comparative mitochondrial membrane potential evaluations of the 3D multicellular structures (see Supplementary Material 2). Both TMRM fluorescence parameters, the TMRM CV and TMRM Ratio (see Materials and Methods), were significantly different in MCF7 spheroids that originated from cells deprived of or exposed to E2 (Figure 4C). While lower TMRM CV and high TMRM Ratio (suggesting relatively high mitochondrial membrane potential and high metabolic activity) characterized MCF7 spheroids grown in the absence of hormone, BC spheroids that were cultured in the presence of E2 showed significantly low TMRM Ratio and higher spatial distribution of the fluorescent signals, reflecting lower metabolic activity. The mean TMRM Ratio was 1.72±0.61 and 0.99±0.38au, p<0.00005, for MCF7 spheroid populations grown in the absence and in the presence of E2, respectively. The mean TMRM CV values for the same spheroid groups were 0.59±0.13 and 0.78±0.27au, p<0.00005, respectively.

The decrease in mitochondrial membrane potential accompanied enhanced cytochrome c release (Figure 4D) as well as elevated caspase-3 activity (Figure 4E, 4F), confirming that mitochondrial apoptotic pathway is induced by hormonal treatment. BC spheroids of both phenotypes were grown, permeabilized and then fixed within the HMA and stained by anti-cytochrome c Ab. Upon exposure to E2, apoptotic cells which have released their cytochrome c from the mitochondria to the cytoplasm, demonstrated significantly reduced staining intensity (see Materials and Methods). Average cytochrome c Fluorescence Intensity (FI) was 8.64±1.4au and 5.7±0.7au p< 1.2^*^10^10^, in estrogen-deprived and E2-exposed spheroid populations, respectively (Figure 4D).

Similar decrease in mitochondrial membrane potential and enhanced cytochrome c release was also observed in ER-positive BC cell line (MCF7:5C) resistant to long-term estrogen deprivation that underwent programmed cell death in the presence of physiologic concentrations of E2 [20].

The activity of caspase-3, a critical executioner enzyme of apoptosis in the caspase cascade, was determined in individual live spheroids, using a bi-functional caspase-3 substrate which consists of a fluorogenic DNA dye and a DEVD moiety. This probe detects real time caspase-3 activity in intact cells. Figure 4E displays caspase-3 activities measured within representative intact hormone-deprived and hormone-treated BC spheroids. While the non-treated BC spheroid population is characterized by low enzyme activity, more than 60% of the E2-treated spheroid population exhibit increased caspase-3 activity. Mean FI signal was 10.05±11.1au and 1.97±1.18au, for E2-treated and E2-deprived spheroids, respectively. p<0.00018 (Figure 4F). The area fraction of caspase-3 activity was respectively significantly higher, following hormonal treatment. (Area fraction of 3.97±6.18% and 24±24.18 in E2-deprived and E2-treated spheroids, respectively. p<0.000004).

The involvement of reactive oxygen and nitrogen species (ROS/RNS) in mitochondria-mediated apoptosis pathways has been widely described [21].

Endogenous NO levels in BC spheroids were measured by vital staining with 4,5-diaminofluorescein diacetate (DAF-2DA) probe, which estimates the relative NO content in cells and spheroids (see Supplementary Material 3). Upon probe loading, the ester bonds of DAF-2DA are hydrolyzed by intracellular esterases, generating DAF-2, which accumulates within the cell, and becomes fluorescent upon oxidation by NO. A significant difference was found in the basal NO content of the two spheroid phenotypes. In E2-treated spheroids which underwent extensive apoptosis, the basal NO content was higher than in hormone deprived clusters (Figure 4F). Average DAF-2 FI was 5.55± 3.21au and 9.65±5.23au p<6.2*10^-9^ in non-treated and E2-treated cell clusters, respectively.

Similar association between elevated NO concentrations and cellular apoptosis was described by others in several tissues including liver [22] and cardiovascular system [23].

**Figure 5:**
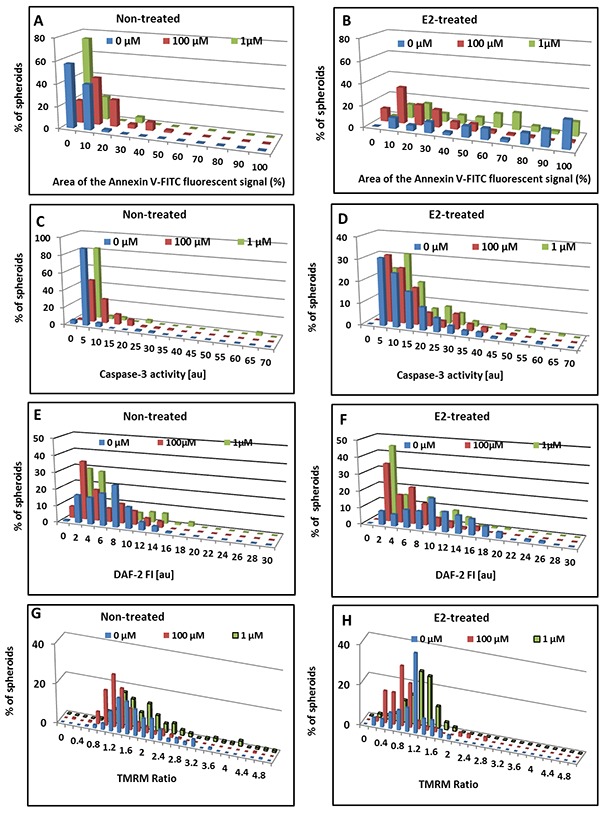
Effect of exogenous NO on apoptotic parameters of MCF7 BC spheroids Distribution histograms of Annexin V fractional area **(A,B)** caspase-3 activity **(C,D)**, relative DAF-2 FI **(E,F)** and TMRM Ratio **(G,H)** in individual MCF7 3D structures upon 24h incubation with DETA/NO. A,C,E,G – spheroids generated from hormone-deprived cells. B,D,F,H – those derived from E2-treated spheroids.

The impact of extracellular NO on apoptosis levels of MCF7 3D structures is shown in Figure 5. Incubation with 1μM NO donor did not provoke any alteration in apoptosis levels in 3D structures deprived of hormonal treatment, since no changes were observed, either in Annexin V signal, caspase-3 activity, or in endogenous NO levels (Figure 5A,5C,5E).

However, 100μM DETA/NO induced significant alterations; 79% of the MCF7 clusters became Annexin V-positive and the average area fraction of the fluorescent signal increased to 10% (Figure 5A). Concomitantly, exposure to 100μM NO donor significantly augmented intracellular caspase-3 activity (Figure 5C) and cytochrome c release, indicating a mitochondrial apoptotic pathway mediated by higher concentrations of NO.

Interestingly, exposure to DETA/NO significantly attenuated E2-dependent phosphatidylserine (PS) externalization in MCF7 spheroids at both donor concentrations. Almost all spheroids cultured in the presence of E2 were Annexin V-positive and showed high variability in the probe FI levels (Figure 5B). NO-donor at 1μM and 100μM decreased the average area fraction of Annexin V signal by 49.3% and 58.5% respectively. The average area fraction of Annexin V-FITC fluorescent signal was 55.8±25.0%, 28.3±24.7% and 23.2±17.9% for control, 1μM and 100μM DETA/NO, respectively, p<0.0005 for both concentrations. Concomitantly, the amount of dead cells, as reflected by the area fraction of PI FI in these hormone-treated spheroids, decreased from 5.01±5.5 % to 2.6±3.8 % (p=0.004) upon 24h exposure to 1μM NO donor.

In addition to the decrease in PS externalization, 24h exposure of hormone-treated spheroids to DETA/NO at both concentrations resulted in significant reduction of endogenous NO levels (Figure 5F). Average DAF-2 FI in this phenotype decreased by 50%.

However, neither cytochrome c translocation from mitochondria to cytoplasm, nor the high enzymatic activity of caspase-3 which characterizes spheroids derived from E2-treated MCF7 cells, were affected following 24h exposure to NO donor at both concentrations. (Figure 5D), demonstrating that this mitochondrial cell death pathway was not inhibited.

As mentioned above, basic metabolic activity of 3D structures is significantly different in MCF7 spheroid phenotypes that were generated from cells exposed to E2, than of those generated from non-treated cells. The influence of exogenous NO on mitochondrial membrane potential in the two types of BC spheroids is depicted in Figure 5G, 5H. In accordance with the impact of NO on spheroid growth, a dual effect of NO on 3D structures deprived of hormonal treatment is evident. Exposure to 1μM NO donor enhanced mitochondrial activity (average TMRM Ratio increased from 1.7±0.4au to 2.1±0.6au, p<0.0005) while 100μM NO donor reduced mitochondrial membrane potential (TMRM Ratio 1.3±0.3au, p<0005) (Figure 5E) concomitantly with the increase in Annexin V signal (Figure 5A), and enhanced caspase-3 activity (Figure 5C).

For E2-treated spheroid phenotype, which exhibited low metabolic activity, 24h incubation with 1μM NO donor slightly increased TMRM staining, and 100μM DETA/NO further reduced mitochondrial membrane potential (Figure 5F). Average TMRM Ratio values were 0.99±0.34au, 1.09±0.33au, and 0.78±0.49au, p=0.01 for spheroids incubated without and with the presence of 1μM and 100μM DETA/NO, respectively.

**Figure 6:**
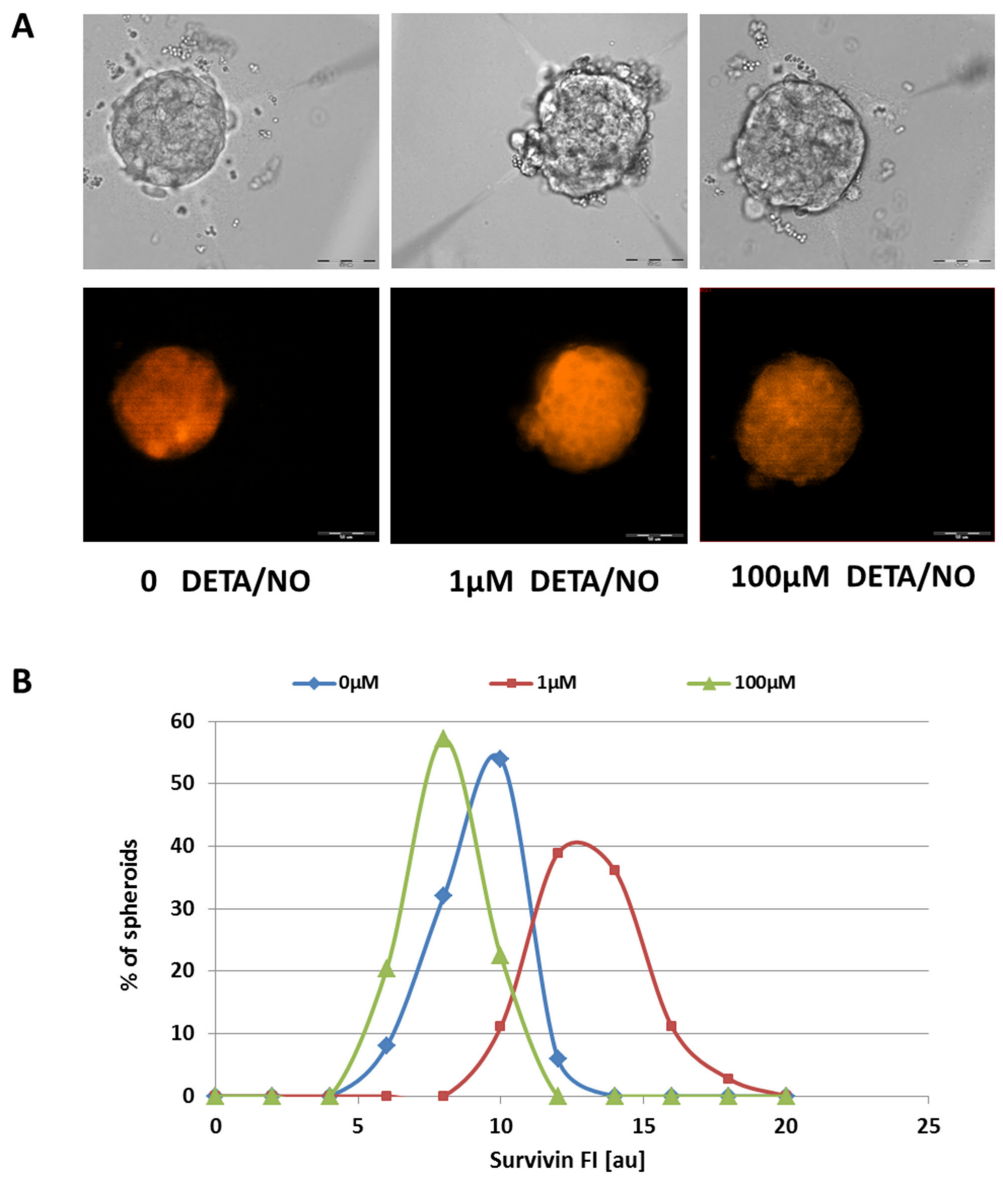
Expression levels of survivin in hormone treated BC spheroids **(A)** Bright field (upper panel) and corresponding fluorescence images (lower panel) of representative E2-treated spheroids upon 24h exposure to 1μM and 100μM NO donor, following fixation and immune-staining for survivin. Scale bar = 50μm. **(B)** Distribution histograms of survivin expression levels in E2-treated individual 3D structures upon 24h incubation with DETA/NO.

Expression of the inhibitor of apoptosis (IAP) protein survivin, in tumors, correlates with inhibition of apoptosis, decreased rate of cell death, aggressiveness and resistance to chemotherapy [24]. In order to investigate the association between this IAP protein and NO effects, intracellular levels of survivin were analyzed by immunofluorescent staining. Two-day BC spheroids treated with hormones were exposed to DETA/NO for 24h and then fixed within the HMC array and stained by anti-survivin Abs. High immunoreactivity of survivin protein was detected in the cytoplasm of the MCF7 spheroids (Figure 6A). Apoptosis suppression of hormone-treated BC spheroids by NO involved modulation of survivin expression, since low doses of NO donor augmented the intracellular levels of the IAP protein (Figure 6B). Mean FI and the area fraction of survivin signal were both significantly elevated in E2-treated spheroids that were exposed to 1μM DETA/NO. Conversely, survivin levels were down-regulated in E2-treated spheroids that were exposed to 100μM DETA/NO (Figure 6B). Average survivin FI in E2-treated spheroid population was 8.05± 1.3au, 12.1± 1.7au, and 6.9± 1.2au, before and after incubation with 1μM and 100μM NO donor, respectively, p<4.5^*^10^5^ for both concentrations.

Overexpression of survivin was shown to be associated with inhibition of cell death initiated either through the extrinsic or the intrinsic apoptotic pathways [25]. It has been shown that survivin and other IAPs can directly or indirectly inhibit caspases or pro-caspases, respectively [26].

Intriguingly, in BC spheroids that undergo estrogen-induced apoptosis, NO-induced upregulation of survivin was not associated with a decrease in caspase-3 activity. This may indicate that under the experimental conditions used here, during the 24h exposure of 3D structures to low doses of NO donor, the anti-apoptotic pathway had already been activated, but the enzymatic activity of the cell death protease had not yet been significantly interrupted.

### Effect of NO on ER expression levels in MCF7 spheroids

**Figure 7:**
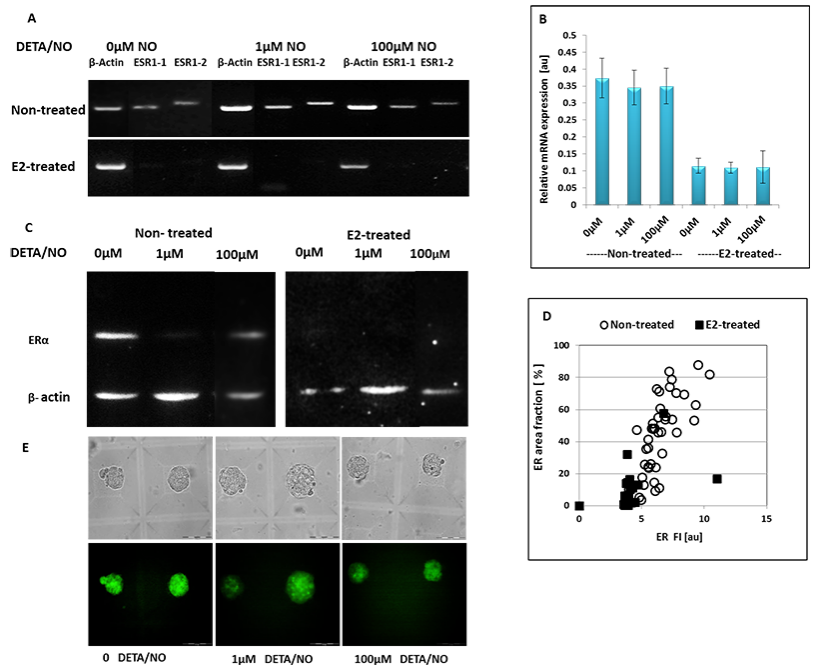
The effect of NO-donor on ER status in hormone-deprived and E2-treated MCF7 spheroids **(A)** Gel visualization of the PCR products. **(B)** Relative mRNA expression levels of ER, calculated as density of the product of ESR1 divided by that of the β-actin from the same cDNA. Bars represent SE of 3 experiments. **(C)** Relative protein expression levels of ER protein. **(D)** Fluorescence parameters of ER staining in individual MCF7 spheroids generated from E2-treated (solid squares) and non-treated cells (hollow circles). **(E)** Bright field (upper panel) and corresponding fluorescence images (lower panel) of two representative spheroids generated in adjacent MCs before and after 24h incubation in the presence of 1μM and 100μM DETA/NO. Scale bar = 100μm.

In agreement with the results shown in 2D culture, ER was downregulated both at the mRNA and protein levels in spheroids generated from hormone-treated MCF7 cells. Upon exposure to estrogen, the 3D structures expressed low mRNA levels (Figure 7A, 7B, 0μM), undetectable ER protein as measured by western blotting (Figure 7C, 0μM), and significantly altered fluorescent signals by immunostaining (Figure 7D). Relative mRNA levels decreased from 0.37±0.03au to 0.11±0.10 au, p<0.0008, following hormonal treatment (Figure 7B).

The immunofluorescent staining of ERα in two representative 3D structures is shown in Figure 7E (left panel). Green fluorescence was not restricted to the nucleus, but was rather scattered throughout the cytoplasm and on cell borders. Hence, ER seems to redistribute from the nucleus into extranuclear sites. In those experiments where the primary anti-ERα antibody was omitted, there was no green staining at all, indicating that the green fluorescence shown in Figure 7E is specific staining of ERα. Using a cutoff value for ER-positive spheroids as the mean FI±SD of control spheroid population (n=100, without primary Ab, see Materials and Methods for ER immunostaining and image analysis), revealed that ER fluorescent signal was lower in E2-treated BC spheroids. While prior to hormonal treatment, the whole MCF7 spheroid population was ER-positive, after E2 treatment, about 82% of the spheroid population was found to be ER-positive. Moreover, the FI and the area fraction of ER fluorescent signal were both significantly lower after hormonal treatment (Figure 7D). The averaged area fraction of the fluorescent signal decreased from 44.5±23.4% to 5.8±10.4% and the mean FI from 6.5±1.4au to 3.5±1.9 au, for cell clusters derived from non-treated and E2-treated cells, respectively, p<0.0005 for both cases.

48h BC spheroids generated from E2-treated and non-treated MCF7 cells were exposed to DETA/NO for 24h and then either fixed within the HMC array and stained by anti-ERα Abs for imaging-based analysis or recovered from the array, pooled and analyzed using reverse transcription polymerase chain reaction (RT-PCR) and western blotting.

**Figure 8:**
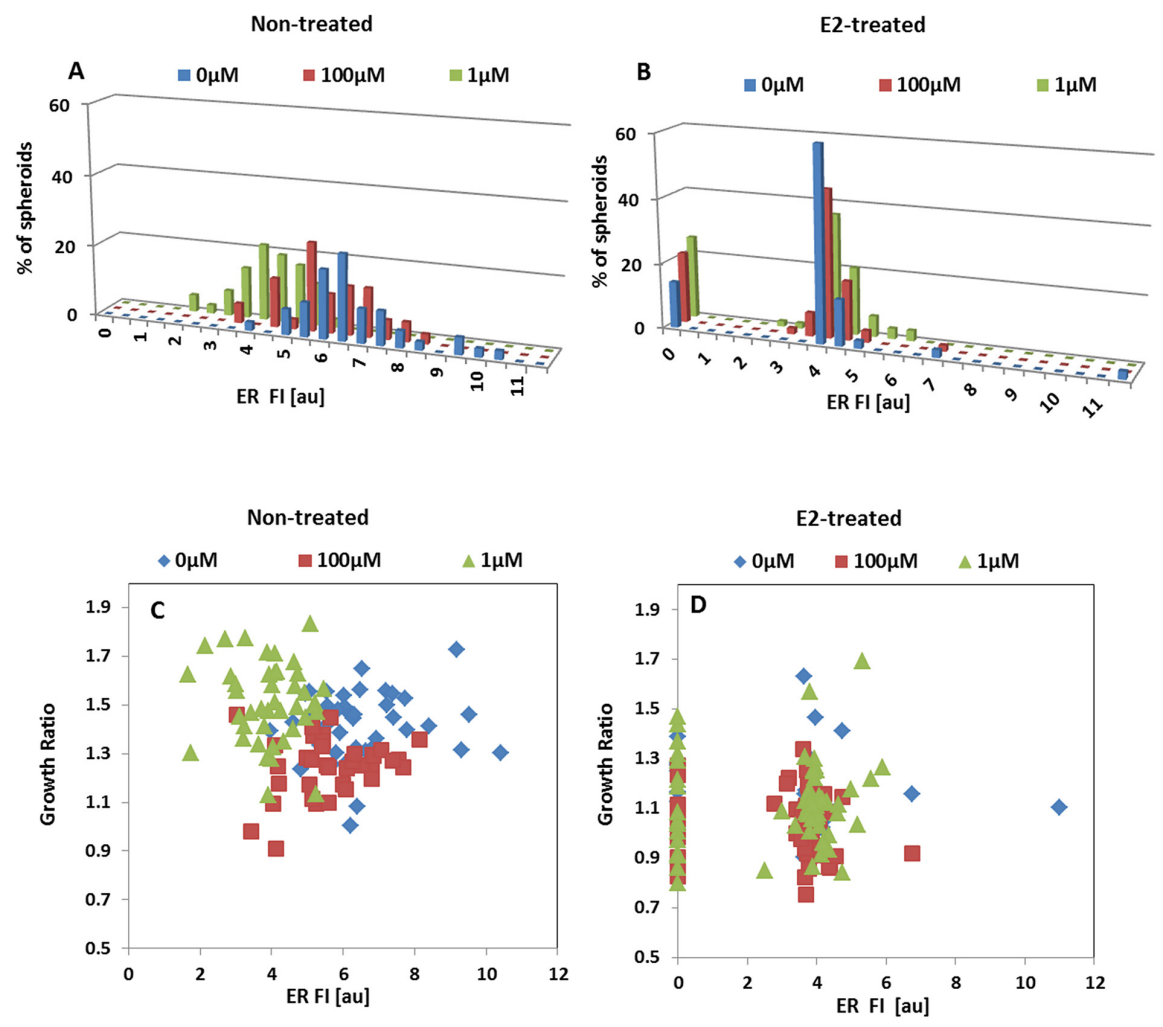
Effect of NO on the relative expression of ER by MCF7 spheroids Distribution histograms of ER expression levels in individual MCF7 3D structures upon 24h incubation with DETA/NO. **(A)** Hormone deprived spheroids. **(B)** E2-treated MCF7 spheroids. Correlation between GR and ER levels in E2-deprived **(C)** and E2-treated **(D)** individual spheroids upon exposure to NO-donor.

ER levels in 3D structures derived from non-treated MCF7 cells were significantly downregulated upon exposure to 1μM NO donor, as measured either by averaging protein content using western blot analysis (Figure 7C) or by image analysis at the resolution of individual spheroids (Figure 8A, 8B). As indicated by western blot, a significant decrease in the relative ER protein level from 1.43±0.02au to 0.54±0.15au was evident after 24h incubation with 1μM DETA/NO, p=0.027, but no change was observed in the presence of 100μM DETA/NO (relative protein level 1.56±0.19au p>0.05).

RT-PCR analysis indicated that the downregulation of ER expression level is post-translational, since ER mRNA did not change upon incubation with NO donor (Figure 7A, 7B).

Similar results were obtained by ER immunostaining (Figure 8A). Both ER fluorescence parameters – spheroid mean FI and the area fraction of the fluorescent signal were significantly lower in BC spheroids treated with 1μM NO donor. The intensity of the fluorescence signal and its area fraction were 6.4±1.4au and 44.5±23.7% respectively, and decreased to 3.9±0.9au and 14.9±14.3% following 24h incubation in the presence of 1μM DETA/NO (p<0.005, for both parameters). However, exposure to higher concentrations of DETA/NO (100μM) did not induce a significant effect on ER expression level in these cell clusters (Figure 8A).

About 15-20% of E2-treated spheroids are ER negative. Nevertheless, in contrast to the effect on E2-deprived phenotype, 24h incubation in the presence of DETA/NO at both concentrations did not induce significant changes on protein levels of the hormone receptor (Figure 8B).

The substantial downregulation of ER induced by low NO concentrations in E2-deprived MCF7 spheroids correlated with the increase in GR parameter (Figure 8C). As seen, the spheroid population that was exposed to 1μM DETA/NO showed a higher proliferation rate and concomitantly exhibited lower receptor levels. Using the K-means algorithm, we were able to cluster the three spheroid sub-groups according to both parameters: GR and ER expression levels. For E2-treated 3D clusters, no such correlation exists (Figure 8D).

Estrogen and progesterone receptors are likely to be lost during hormone therapy [27]. Moreover, the loss of ER may reflect a mechanism that can result in endocrine resistance [28].

Hence, the substantial downregulation of ER induced by low NO concentrations concomitantly with an increase in GR and in TMRM Ratio, may imply a shift into more aggressive BC phenotypes.

### Effect of NO on spheroid invasion

**Figure 9:**
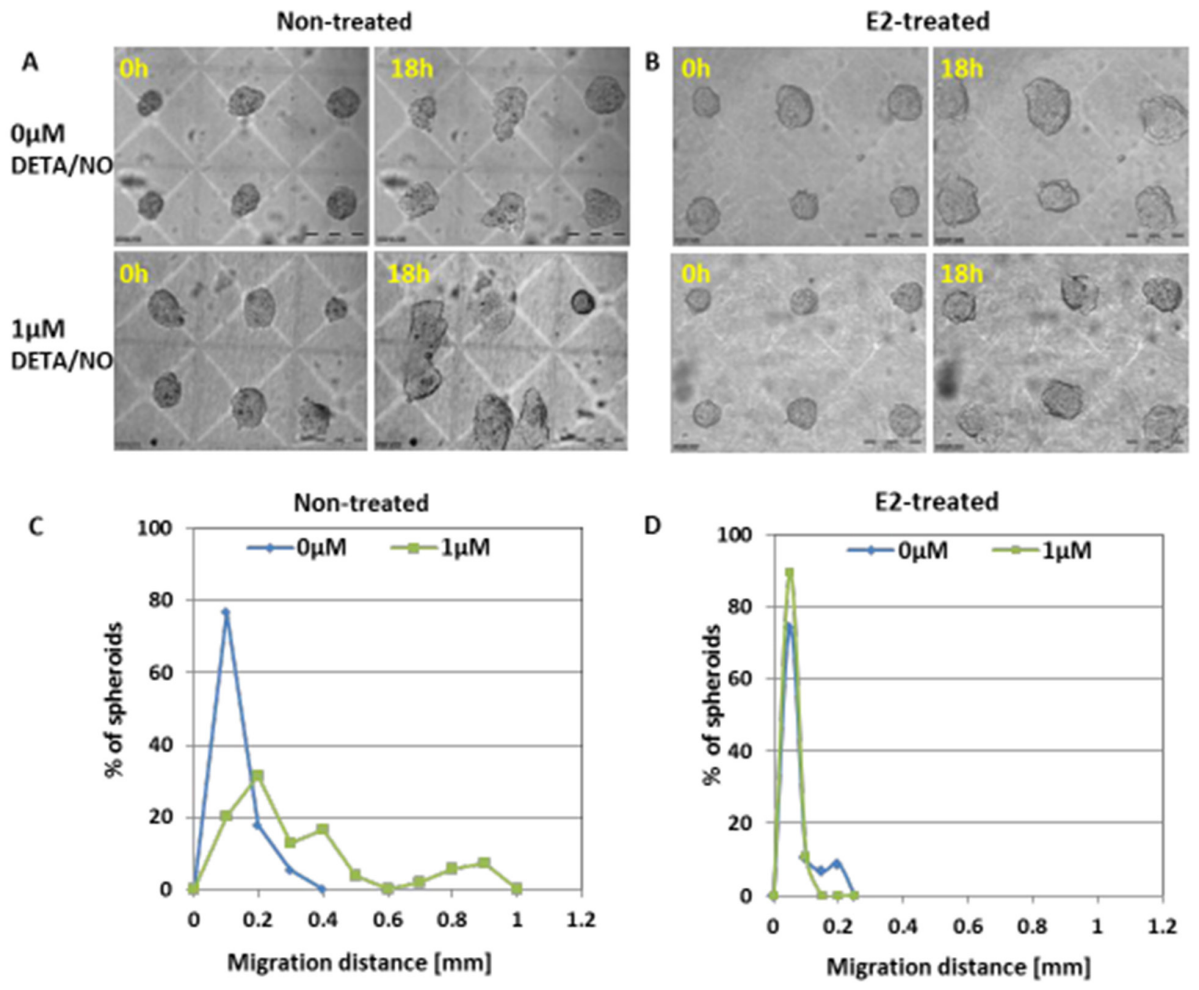
Invasion capacity of MCF7 BC spheroids Changes in morphology and location of representative non-treated **(A)** and E2-treated **(B)** 2-day BC spheroids embedded within collagen matrix and cultured for 18h in the presence and in the absence of 1μM DETA/NO. Scale bar = 200μm. Distribution histograms of the averaged migration distance of hormone-deprived **(C)** and E2-treated **(D)** BC spheroids over an 18h period in collagen matrix in the presence and in the absence of 1μM DETA/NO.

**Figure 10:**
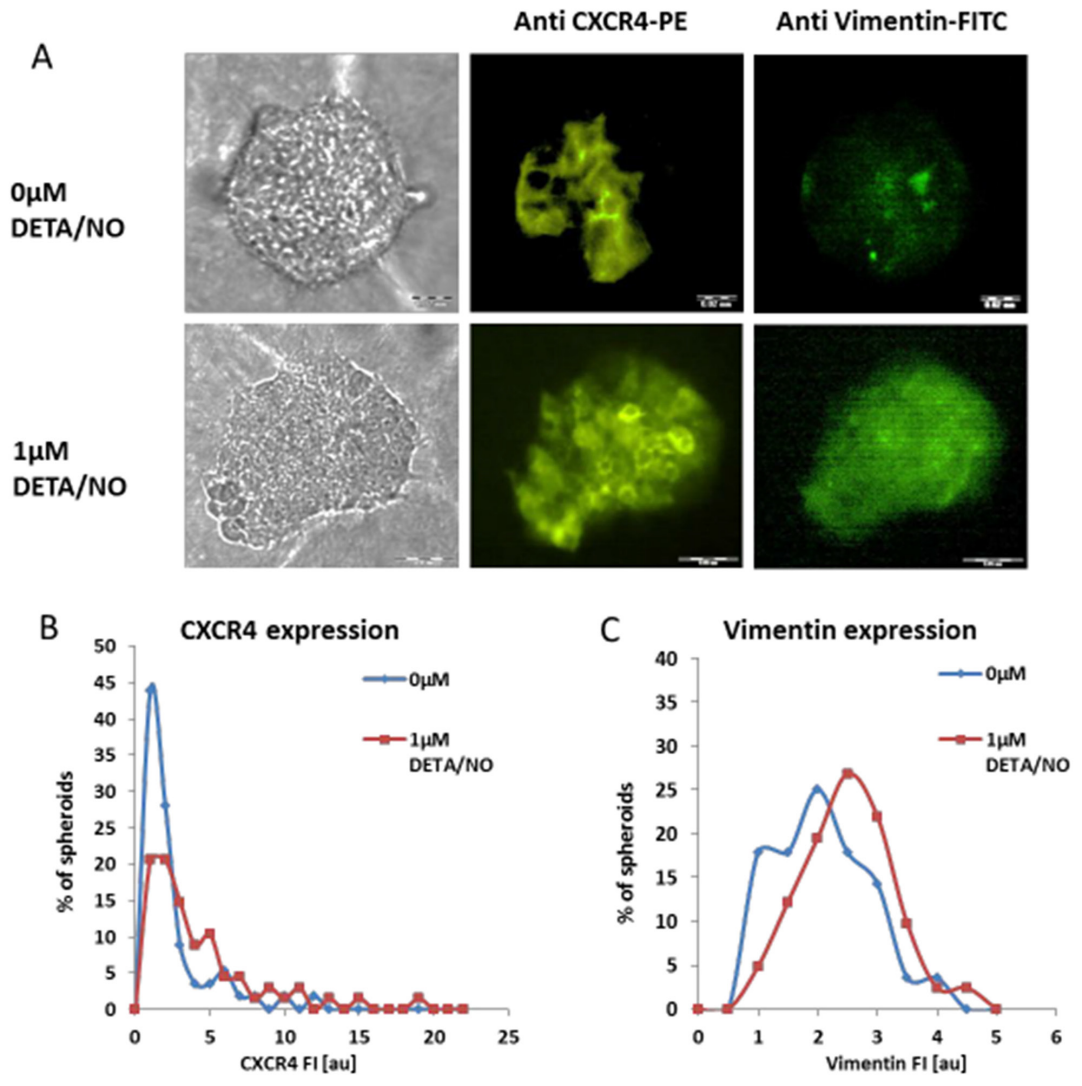
Expression levels of cellular proteins upon NO-induced migration Hormone deprived BC spheroids were embedded within collagen matrix and cultured for 24h in the absence (upper panel) and in the presence (lower panel) of 1μM DETA/NO. Then, live spheroids were stained by anti CXCR4 Abs, followed by fixation, permeabilization and additional staining by anti-vimentin Abs. **(A)** Bright field and corresponding fluorescence images of representative 2-day BC spheroids double stained for CXCR4 and vimentin are shown. Scale bar = 200μm. Distribution histograms of CXCR4 **(B)** and vimentin **(C)** expression levels in individual hormone deprived MCF7 3D structures upon 24h exposure to DETA/NO.

It has been shown that low NO flux may stimulate tumor expansion and migration/invasion [29], hence, the possibility that low NO concentrations induce an aggressive invasive phenotype, was tested. MCF7 spheroids were embedded within the extra-cellular matrix (ECM) component and the migration dynamics of each cell-cluster was measured in response to 1μM DETA/NO. Changes in morphology and location of 3D spheroids over 18h period of exposure to NO donor, is shown in Supplementary Videos 3-6 and Figure 9. Upon embedding in collagen, both MCF7 spheroid phenotypes remain spherical cell aggregates with smooth surfaces. In the course of culturing within the collagen matrix, spheroids tend to lose their sphericity (Figure 9A, 9B upper panel). Interestingly, in the presence of 1μM DETA/NO, hormone-deprived MCF7 BC spheroids dramatically transformed their shape and position (Figure 9A). These spheroid phenotypes become more flat and translocate in the surrounding collagen matrix. Before exposure to NO donor, the average migration distance in collagen over an 18h period, was 78.8μm (n=100) while in the presence of DETA/NO, MCF7 spheroids exhibited heterogeneous invasion capacity and the average migration distance was significantly extended (316.4μm, p<0.0005) (Figure 9C). Conversely, E2-treated cell clusters, did not move within collagen matrix, and no evidence of collective invasion/migration was found upon exposure to 1μM NO donor (Figure 9B). Migration distance was 43.1μm and 33.2μm in the absence and in the presence of DETA/NO, respectively p>0.05 (Figure 9D). The collective invasion induced by NO in E2-deprived spheroids was accompanied by over-expression of two cellular proteins; CXCR4 and vimentin. CXCR4, a G protein-coupled cell surface chemokine receptor, was shown to promote BC metastasis to organs [30]. Typical membrane staining is observed upon vital probing of BC spheroids by anti CXC4R Abs. (Figure 10A). The averaged FI signal of CXCR4 protein in live BC spheroids increased from 1.98±2.3au to 4.24±5.0 (p<0.0013), and the area fraction of the receptor signal increased from 7.8±7.1 au, to 16.4±18.1, p<0.0008, following NO mediated migration (Figure 10B). Concomitantly, the same spheroids which exhibited an invasive phenotype, showed increased vimentin levels (Figure 10C). Averaged vimentin FI signal was 1.82±0.7 au and 2.24±0.76 au, (p<0.03), before and after exposure to NO donor, correspondingly. However, no correlation was found between the cellular expression levels of CXCR4 and vimentin, measured on the same cell clusters (Pearson correlation = -0.03).

Overexpression of vimentin, a major constituent of the intermediate filament of proteins, was shown to be correlated with aggressive phenotype and increased invasion of BC cell lines [31]. Furthermore, vimentin was reported to play a major role in the epithelial-to-mesenchymal transition (EMT) process of BCs, and its knock-down resulted in a decrease in genes linked with BC invasion and the basal-like phenotype [32]. Collective migration is characterized by the migration of whole groups of cells interconnected by adhesion molecules and other communication junctions [33]. A hybrid epithelial/mesenchymal (hybrid E/M) phenotype is associated with this type of migration. Although an increase in vimentin, a requisite regulator of mesenchymal cell migration, was reported here, more tests are needed in order to comprehend the EMT status of NO-induced invasive BC phenotype.

## DISCUSSION

It is estimated that during the next 15 years there will be a 50% increase in the incidence of BC, mainly in ER-positive, which will become 95% of this disease, while only 5% will be ER-negative [34]. Strategies to prevent or cure ER-positive BC are an important priority in healthcare. The biology of estrogen-induced apoptosis has created a need for therapeutic approaches to block cell survival pathways in order to augment hormone-induced apoptosis and to provide an inexpensive targeted therapy to maintain ER-positive BC patients indefinitely. However, the lack of biomimetic *in vitro* models that can recapitulate the features of solid tumors impedes the development of such new therapies. In order to overcome the significant distinctions in drug sensitivity between conventional 2D cell culture and 3D tumor multi cellular structures [35], as well as the substantial differences that exist between rodent and human NOS and the NO fluxes [36], a sensitive human BC spheroid model was used in the current study. This unique 3D culture system is utilized for the first time, to assess NO effects on hormone vulnerable BC cells. Using this cellular model, we were able to demonstrate that low doses of NO-donor induce cell proliferation, high metabolic activity, downregulation of ER and collective invasion in BC spheroids that are sensitive to estrogen-induced apoptosis, thus contributing to more aggressive phenotypes. Moreover, following hormone addition, 3D BC clusters were rescued from E2-induced apoptosis by these low concentrations of NO-donor, since NO attenuated the level of PS externalization, cell death and increased metabolic activity. NO cytoprotection involves modulation of the IAP protein, survivin, since low doses of the NO-donor considerably upregulate its expression.

Collectively, the results may suggest that NO, in nanomolar concentrations, is an inhibitor of estrogen-induced apoptosis and thus, plays a major role in this hormonal therapy.

In line with the above results, Fetz et al [37] showed that low NO levels conferred survivin-mediated resistance against cisplatin/taxol-induced apoptosis in head and neck squamous cell carcinoma lines. Additionally, endogenous NO was shown to be involved in tamoxifen-resistance in ER-positive BC [38], since it was essential for the completion of autophagy and the protection of ER^+^ MCF7 BC cells from tamoxifen-induced cytotoxicity.

Importantly, in MCF10A, a non-tumorigenic mammary epithelial cell line, estrogen-induced apoptosis mediated by extranuclear ERs was blocked by NO via the cGMP pathway [39]. This may suggest that BC cells use existing mechanisms that occur in the normal breast epithelium, in addition to tumor-specific mechanisms which arise from genetic alteration such as chromosomal instability and mutated enzymes.

Furthermore, human BC lines, and especially the MCF7 line, are not single entities, but are rather comprised of large numbers of individual phenotypes that differ in gene expression profile, receptor expression and signaling pathway usage. Dominant phenotype proportions may be maintained by growth conditions such as the presence of hormones. This heterogeneity is likely attributed to all human BC cell lines, as well as to human BCs growing *in vivo* [40], and probably affects the overall response. The HMC array methodology described herein facilitates formation, treatment and monitoring of individual multicellular structures from a distinct small number of cells seeded in each MC, thus enabling analysis at single element resolution and, as a result, a high assay sensitivity. Due to the latter, specific sub-populations of spheroids that would go undetected in population-based measurements were revealed, allowing the study of tumor heterogeneity. Although not in the scope of this work, HMC technology also promotes retrieval of specific spheroids for molecular study of the different pathways that occur during NO regulation of BC growth.

In the current study, low doses of DETA/NO considerably downregulated the expression levels of ER within a period of 24h, probably through the post transcriptional process. Modulation of gene expression by NO has been demonstrated by others either indirectly by inhibiting NOS [17], or directly by using NO releasing agents [41]. Moreover, histone posttranslational modification was found to be the mechanism that supports gene expression modulation by NO [42]. Nevertheless, to the best of our knowledge, regulation of ER gene expression by NO has not been reported yet.

NO is a versatile and pleiotropic molecule. Generation of NO by its various NOSs in normal and malignant tissue is involved in cell signaling, and regulates a variety of physiological functions including cell survival, differentiation and death [43]. In cancer, the biological effects of NO are based not only on tumor type, but are mainly governed by their cellular concentrations along with spatial and temporal distribution both within tumor cells and in the tumor microenvironment.

At low concentrations (<100nM), NO has been reported to manifest a pathological phenotype characterized by increased proliferation, invasion and metastasis, stimulation of angiogenesis and inhibition of apoptosis. At super-physiological concentrations (<400nM), a less aggressive phenotype is evident, where metastasis and angiogenesis are inhibited, and apoptotic machinery operates appropriately [43, 44].

Utilizing a 3D culture system, the current study uniquely demonstrates a biphasic effect of NO on a specific phenotype, vulnerable to estrogen-induced apoptosis. In hormone deprived BC spheroids, NO, at a nanomolar level, stimulated tumor progression and invasion; while higher NO concentrations (100nM) inhibited cell growth and metabolism and promoted mitochondrial mediated apoptosis. Following exposure to estrogen, a low dose of NO-donor represses apoptosis, while higher NO concentrations induce mixed effects on hormone treated BC phenotype; reduced proliferation and metabolic activity, as well as downregulation of survivin levels on the one hand, with inhibition of PS externalization on the other.

NO concentrations and their influences upon tumor cells are complicated by threshold amounts. Based on several reports of NO measurement in tissues, and from exogenous NO donors, it has been established that normal NO levels are generally below 50nM [44, 45]. The estimation of NO levels in hormone susceptible MCF7 spheroids showed compatible results. The basic difference in NO content found in the two BC phenotypes may account for at least some of the differences in their response to exogenous NO, since it has been demonstrated that select signal transduction cascades respond to NO exposure with different threshold sensitivities, resulting in distinct phenotypic reactions. Hence, Thomas D et al [46] showed that low NO doses of 10-300 nM induced ERK phosphorylation and HIF-1α (hypoxia induced factor 1α) accumulation in MCF-7 cells, resulting in tumor proliferation and differentiation, while with high doses of NO (> 300 nM), p53 phosphorylation occurs, which is associated with apoptosis induction [46].

Interestingly, inhibition of hormone-induced apoptosis and other cyto-protective actions occur here, at the very low (∼1nM) NO level.

It is now widely recognized that many tumors utilize low-flux NO to resist apoptosis, stimulate expansion through accelerated proliferation and migration/invasion, and to resist eradication by anti-tumor therapies such as PDT as well. This low-flux NO can be generated by tumor cells per se, but surrounding vascular cells such as eNOS-expressing endothelial cells may also be a contributing factor in their generation [29].

These negative and potentially tumor-promoting side effects of NO, at least in PDT and possibly in other therapeutic strategies, may be averted through use of iNOS inhibitors as adjuvants [47].

The role of hyponitroxia (persistently low NO concentrations) as a key mediator in tumor progression has recently been discussed [48] and may become a novel therapeutic target to treat cancer and provide new opportunities for pharmacological intervention [49].

## MATERIALS AND METHODS

### Materials

Low-melt agarose (LMA) was obtained from Cambrex Bio Science Rockland, Inc. (Rockland, ME USA). LMA was dissolved in phosphate buffer saline (PBS) at room temperature (RT) (stock solution concentration 6%) and sterilized in an autoclave. Sylgard 184 Kit was purchased from Dow Corning Corp. (Midland, MI, USA).

Six or 24 microwell glass bottom plates were purchased from *In Vitro* Scientific (Sunnyvale, CA, USA).

RPMI, medium, Dulbecco’s Modified Eagle’s Medium (DMEM), heat-inactivated fetal calf serum (FCS), penicillin, streptomycin, sodium pyruvate and PBS were obtained from Biological Industries (Kibbutz Beit Haemek, Israel). DiethylenetriamineNONOate (DETA/NO) was obtained from Enzo Life Sciences (Farmingdale, NY, USA) and the stock solution (10 mM) in PBS was kept at -80°C.

Anti-ERα and anti-β-Actin mouse monoclonal IgG antibodies, secondary goat anti-mouse IgG-HRP and IgG-FITC, and collagen type 1 (rat) and 4,5-diaminofluorescein diacetate (DAF-2DA) were obtained from Santa Cruz Biotechnology (Santa Cruz, CA, USA). Anti-survivin-PE, anti-CXCR4-PE and anti-cytochrome c-FITC human monoclonal IgG antibodies were purchased from Miltenyi Biotec (Germany). Collagen solution (3mg/ml) was prepared on ice and the pH was adjusted to 7.4 using NaOH, PBSx10 and sterile DDW according to manufacturer’s instructions.

17β-estradiol (E2), propidium iodide (PI), tetramethylrhodamine methyl ester perchlorate (TMRM), Ponceau S solution and Nω-nitro-L-arginine methyl ester (L-NAME) were purchased from Sigma-Aldrich Israel Ltd. (Rehovot, Israel). Hoechst 33342 dye trihydrochloride, trihydrate was obtained from Invitrogen-Molecular probes (Carlsbad, CA, USA). Apoptotic cells were detected using Annexin V-FITC Apoptosis Detection Kit (BioVision, USA) and NucView™ 488 Caspase-3 Assay Kit (Biotium, Inc, Fremont, CA, USA).

Lowry assay kit and SuperSignal™ West Pico Chemiluminescent Substrate were purchased from ThermoFisher Scientific (USA).

Design and fabrication of hydrogel array and imaging device, as well as cell loading and spheroid generation are described in Supplementary Material 4.

### MCF7 cell line vulnerable to E2-induced apoptosis

MCF7 human BC cell line was cultured for more than 5 years in DMEM with 10% heat-inactivated FCS, 100 U/mL penicillin, 100 μg/mL streptomycin, 2% glutamine, 2% sodium pyruvate (DMEM complete medium) without hormonal supplementation. The cells were incubated under standard cell culture conditions at 37°C, 5% CO_2_ in humidified incubators. For E2 treatment, MCF7 cells were maintained in DMEM complete medium supplemented with 10^-9^M E2 for 5 weeks, E2 stock was in 100% ethanol, final concentration of ethanol was 0.002%. E2-treated cells were used in experiments after 5-8 passages.

### DETA/NO treatment

Following 48h of growth, the spheroids were exposed to DETA/NO (0.5-200μM) in DMEM for 24h at 37°C in a CO_2_ humidified incubator. DETA/NO decomposes spontaneously in aqueous media, releasing two mol of NO per mol of parent compound. Under conditions used here (pH 7.4 and 37°C), the half-life of DETA/NO is about 24h. The amount of NO generated from DETA/NO in DMEM was measured [50] and the steady state concentrations of NO estimated at about 100nM and 1nM for 100μM and 1μM DETA/NO respectively [50].

### Fluorescent staining

Mitochondrial membrane potential was measured by TMRM staining. For in-HMC array TMRM staining, the medium was removed from the macro-well and replaced with the same volume of medium containing 12.5nM TMRM. Spheroids were incubated for 1h at 37°C under humidified atmosphere with 5% CO_2_, then washed twice, and imaged. For quantitative analysis of the overall mitochondrial membrane potential of 3D spheroids under experimental conditions, the same spheroid population was stained twice at two time points, before and after 24h treatment, and imaged. The following parameters were obtained: TMRM Fluorescence Intensity (FI); the averaged FI of all the pixels within a spheroid area, TMRM CV; the coefficient of variation (CV) of pixel intensity within a spheroid area and TMRM ratio; the ratio between TMRM FI of a spheroid at two time points. A detailed analysis of TMRM fluorescence parameters for mitochondrial membrane potential is described in Supplementary Material 2.

Cell nuclei were stained with Hoechst 33342 dye by incubating the spheroids in the presence of the probe at final concentration of 1μg/mL for 1h at 37°C under humidified atmosphere with 5% CO_2_ followed by washing and imaging. For in-HMC array double (Hoechst 33342 and TMRM) spheroid staining, the entire medium was removed and exchanged with medium containing both probes in their final concentrations.

To assure uniform cell staining across the 3D cell clusters under the above conditions, images were acquired in 8μm increments along the spheroid z axis, and the mean FI of all pixels within each focal plane were calculated. For both probes (TMRM and Hoechst 33342), the CV of mean FI (CV FI) of the different focal planes (n=100 spheroids) did not exceed 8±2.6%, indicating homogeneous staining of the 3D objects.

Apoptosis was evaluated by Annexin V test. The entire medium was removed and 50μL of binding buffer (HEPES buffer, pH 7.4) with Annexin V-FITC (final concentration of 1mg/ml) was added to spheroids within the MCs for 15min in the dark at RT. The average FI of all pixels within a spheroid area was calculated. The cutoff value for Annexin V-positive spheroids was mean FI+SD of control unstained spheroid population (n=100).

In order to dye the same spheroids with both TMRM and Annexin V probes, cell clusters were successively stained, first with TMRM (as described above), then washed twice and stained with Annexin V-FITC.

For the estimation of the relative intracellular NO concentration in 3D spheroids, the medium was removed and replaced with the same volume of medium containing 10μM DAF-2DA for 30min at 37°C in a CO_2_ humidified incubator. Then, spheroids were washed by exchanging the medium twice, and imaged. The FI of individual 3D spheroids were extracted by image analysis and the population averaged intracellular FI was calculated.

For control measurements, the DAF-2DA staining was performed in the presence of the NOS inhibitor L-NAME (10μM). A detailed analysis of NO content by DAF-2DA probe is described in Supplementary Material 3.

Caspase-3 activity was evaluated by exposing 3D clusters to NucView 488 substrate (5μM, 90min) at 37°C in a CO_2_ humidified incubator, followed by imaging. The average FI within each spheroid was then, calculated.

### Immunofluorescent staining

Immunofluorescence staining of ER and survivin was carried out following spheroid fixation within HMC array. Upon completion of vital measurements, the entire medium was removed from the macrowell and the spheroids were fixed within MCs by adding 4% formaldehyde for 1h at RT. Then, the cells were permeabilized for 15min at RT with 0.1% Triton 100 in PBS, washed with PBS, and blocked with 4% BSA in PBS for 1h at RT. Fixed spheroids were washed again, incubated in PBS containing anti-ERα mAb (1:100) for 24h at 4°C, and then with FITC-conjugated anti-mouse IgG (1:100) for 3h at RT. Direct staining by anti-survivin-PE mAb (1:10) was performed by incubation at 4°C for 24h.

The quantification of the release of cytochrome c from mitochondria into the cytosol on a per-spheroid basis was done as previously described [51]. Spheroids within HMC array were first permeabilized for 15min by 0.1% saponin, a cholesterol-removing agent that affects the plasma membrane without disrupting mitochondrial membranes [52]. Then, the spheroids were fixed, blocked and directly stained by cytochrome c-FITC mAb (1:100) as described above.

Surface expression level of CXCR4 was evaluated in live spheroids. BC clusters were incubated in 4% BSA in PBS for 1h at RT, followed by direct staining with anti-CXCR4-PE mAb (1:10) for 2h at 4°C. At the end of the staining process, spheroids were washed twice with 0.5% BSA in PBS.

### Tumor cell invasion assay in 3D BC spheroid model

For analysis of invasion capacity, MCF7 BC spheroids were embedded in an extracellular matrix composed of type I rat tail collagen. Two-day BC spheroids generated within HMC array-based plate, were cooled on ice for 10min, and embedded in collagen by replacing the entire medium above the array with a cold collagen solution (150μl). Then, the plate was transferred to a tissue culture incubator for 1h, to promote collagen gelation. Following collagen gelation, 300μl of the warmed (37°C) 1% LMA was poured on top of the collagen gel and the array was incubated first at RT for 5-7min and then for 2min at 4°C, for agarose gelation. The agarose layer prevents detachment of the collagen-gel matrix. Finally, 1-3ml of warm cell culture medium, with or without invasion modulating compounds, was added.

### Western blot analysis

Hormone-treated and untreated MCF7 cells and spheroids were collected, washed twice with ice-cold PBS, lysed in lysis buffer (10mM Tris-HCL, pH=7.4, 1mM EDTA, 0.5mM EGTA, 150mM NaCl, 1% Triton X-100, 0.1% SDS, 0.1% Na deoxycholate, 1mM protease inhibitor PMSF) for 30min on ice and centrifuged at 14000g for 15min at 4°C. Protein samples (20-50μg in different experiments) were separated by sodium dodecyl sulfate-polyacrylamide gel electrophoresis (SDS-PAGE) and transferred to nitrocellulose membrane. The membranes were stained with Ponceau S, blocked with 5% non-fat milk in PBS, 0.05% Tween 20 (PBST) for 1.5h at RT and incubated with the primary ERα antibody (1:500) at 4°C overnight. The membranes were then rinsed in PBST and incubated with secondary IgG-HRP antibodies (1:5000) for 3h at RT. After washing, proteins were detected using SuperSignal™ West Pico Chemiluminescent Substrate Membranes were reprobed with anti-Actin antibodies (1:500).

Background signal was subtracted from the images acquired by western chemiluminescence machine (ImageQuant LAS 4000 mini, GE Healthcare, USA) and the bend area was measured using intensity threshold. All signals above background plus SD were counted.

## RT-PCR

Total cellular RNA from 3-day MCF7 spheroids, was extracted with a TRI Reagent® RNA extraction kit, Sigma-Aldrich Israel Ltd. (Rehovot, Israel), according to manufacturer instructions. RNA pellet was dissolved by ultra-pure water. Total RNA was subjected to PerfeCTa DNase I of Quanta Biosciences Inc. (Beverly, MA, USA) in order to eliminate genomic DNA from samples. For RT reaction, 1μg of genomic DNA-free RNA from each sample was used to synthesize cDNA, by using qScript cDNA Synthesis Kit, Quanta Biosciences Inc. (Beverly, MA, USA) according to manufacturer instructions. Oligonucleotide primers for PCR were designed using Primer3 software and synthesized by IDT (Leuven, Belgium) are shown in Table 1:

**Table 1:** Oligonucleotide primers used for PCR

Target genes	Primer sequence 5’------------3’		Product size (bp)
	Forward	Reverse	
β-actin	ACTCTTCCAGCCTTCCTTCC	CCTGCTTGCTGATCCACATC	302
ESR1-set 1	AGACATGAGAGCTGCCAACC	GCCAGGCACATTCTAGAAGG	299
ESR1-set 2	TGAAGTGCAAGAACGTGGTG	AGCAAGCAAATGAATGGCCA	357

cDNA (1/7) were taken for PCR amplification, 0.4μM of each primer at a final volume of 25μl, using GoTaq G2 Green Master Mix from Promega Corporation (Madison, WI, USA). PCR program is as follows: after initial denaturation at 94°C for 2min, 30 cycles of denaturation (at 94°C for 45s), annealing (at 59°C for 30s) and extension (at 73°C for 30s), a final extension was performed for 5min. Visualization of the RT-PCR products was done using 1.5% agarose gel stained with SYBR™ Safe DNA Gel Stain, Thermo Fisher Scientific Inc. (USA). Gel images were obtained using DNR Bio Imaging System MiniLumi UV-image analyzer (Ma’ale HaHamisha, Jerusalem, Israel), and the densities of the products were quantified using ImageJ analysis software. The relative expression levels were calculated as the density of the product of ESR1 divided by that of the β-actin from the same cDNA.

### Image acquisition and analysis

Imaging system and operating software have been previously described (see Supplementary Material 4 for details). For optical data acquisition and analysis, each set of image acquisitions commenced with acquiring the bright field image of a chosen view field, followed by the acquisition of several fluorescent images, one for each fluorescent probe, taken at different preset time points. A series of regions on the array was chosen and the motorized stage configured to stop at these locations/positions. The initial cell distribution in these regions was imaged and the MC walls maintained the position of each group/individual cell within its MC, guaranteeing that these stage positions represented the same individual groups of cells throughout the experiment. For continuous monitoring of the spheroid formation process, and the course of NO treatment as well, the imaging system was programmed to take images of each saved position automatically at consistent time intervals, and the six-well plate was either left on the microscope stage or placed in an external incubator and then returned to the microscope, verifying that the same regions were scanned, and images of the same spheroids taken.

Spheroids were automatically defined as objects or regions of interest (ROIs) by Sobel edge detection algorithm, and their sectional area was outlined on the bright field image. Then, on each fluorescent wide field image, ROIs were determined by mapping those outlines on the interrogated fluorescent field image. Next, the fluorescent background, determined by averaging the FI detected by camera pixels found between the outlined regions, was subtracted from the fluorescent image. It should be emphasized that determination of background signal and its subtraction from the fluorescent image were performed separately for each of the acquired fluorescent field images. Then, the fluorescent images underwent thresholding, and two parameters were calculated: the mean FI value obtained for each ROI (mean FI of all pixels within a ROI that are within the threshold borders) and the area fraction of the fluorescent signal (area percentage of all FI-positive pixels of a ROI, relative to the area of a ROI) were calculated using Cell^P software.

Morphometric parameters of spheroids were extracted by several image-processing algorithms based on probe-free bright-field microscopy. These include automatic algorithms for MC identification, spheroid segmentation, sphericity or ‘roundness’ of a spheroid and texture-based distinction like smoothness value (e.g. entropy, SD and range of gray values). Size and volume estimation of individual cells and spheroids, as well as the number of cells that comprise each spheroid and calculation of doubling time were done as described in Supplementary Material 1.

For analysis of the 3D structure collective migration, the X and Y coordinates of the center of each ROI/spheroid were determined by Cell^P software at two time points, before (X1,Y1) and after (X2,Y2) invasion assay. Then, the distance (d) of spheroid migration was calculated as d=√((X1-X2)^2^+(Y1-Y2)^2^). The K-means algorithm was used for clustering cells groups.

### Statistical analysis

Each test was performed in duplicate (2 macrowells). Fifteen to thirty images (×10 magnification) from different areas within the array were acquired, yielding about 200 individual MCs and the corresponding spheroids per single macro-well. Although the variability of both the morphology and fluorescence parameters within spheroid populations was very high, most of the measured parameters showed normal distribution. The mean and standard deviation (SD) for each measured parameter was calculated for the various spheroid/cell populations under investigation.

Comparison between groups was performed using the t-test, ANOVA and MANOVA for groups with Gaussian distributions, or non-parametric tests (Wilcoxon, Kruskal-Wallis and van der Waerden) for small groups. A paired two sample for means t-test was used to compare two groups of cell-clusters before and after treatment. Statistical significance of differences was determined at p<0.05.

## SUPPLEMENTARY MATERIALS MATERIALS AND VIDEO























## Abbreviations

BC: breast cancer
CV: coefficient of variation
DETA/NO: diethylenetriamineNONOate
DMEM: Dulbecco’s Modified Eagle’s Medium
E2: 17β-estradiol
ECM: extra-cellular matrix
EMT: epithelial-mesenchymal transition
ER: estrogen receptor
FCS: fetal calf serum
FI: Fluorescence Intensity
GR: growth ratio
HMC: hydrogel microchamber
HP: high-proliferating
IAP: inhibitor of apoptosis
LMA: low-melt agarose
LP: low-proliferating
MC: microchamber
NO: nitric oxide
NOS: NO synthase
PBS: phosphate buffer saline
PBST: PBS, 0.05% Tween 20
PDMS: polydimethylsiloxane
PI: propidium iodide
PS: phosphatidylserine
ROI: region of interest
RT: room temperature
RT-PCR: reverse transcription polymerase chain reaction
SD: standard deviation
TMRM: tetramethylrhodamine methyl ester perchlorate
