# Supplementary material for: Nitric oxide is cytoprotective to breast cancer spheroids vulnerable to estrogen-induced apoptosis
Source: Oncotarget. 2017 Oct 7;8(65):108890–911. doi: 10.18632/oncotarget.21610 (PMC5752490; doi:10.18632/oncotarget.21610)
Supplement: Supplementary file 1 [file oncotarget-08-108890-s001.pdf]

Nitric oxide is cytoprotective to breast cancer spheroids vulnerable to estrogen-induced apoptosis

SUPPLEMENTARY MATERIALS

Supplementary Material 1: Size and volume estimation of individual cells and spheroids and Calculation of doubling time.

See Supplementary Material 1

Supplementary Material 2: TMRM staining and mitochondrial membrane potential analysis of 3D spheroids.

See Supplementary Material 2

Supplementary Material 3: Estimation of intracellular NO concentration using DAF-2DA indicator.

See Supplementary Material 3

Supplementary Material 4: Design and fabrication of hydrogel array and imaging device, Cell loading and spheroid generation and Imaging system and operating software.

See Supplementary Material 4

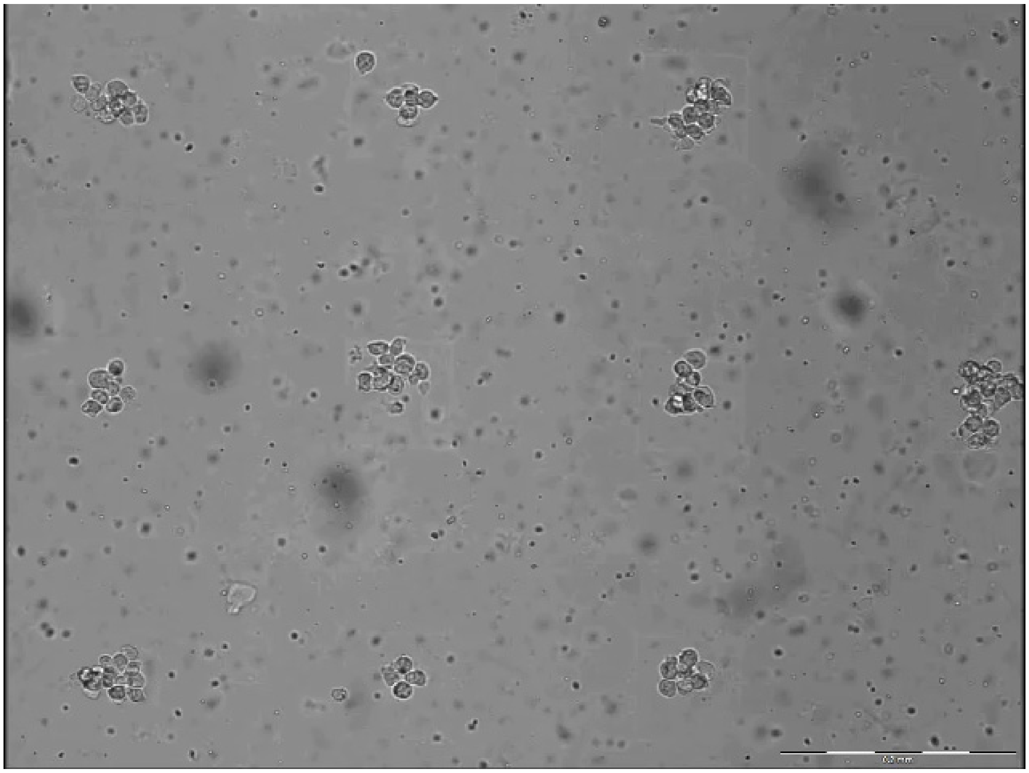

Supplementary Video 1: Spontaneous creation of 3D multicellular spheroids within hydrogel array from (A) Non-treated MCF7 cells.

See Supplementary Video 1

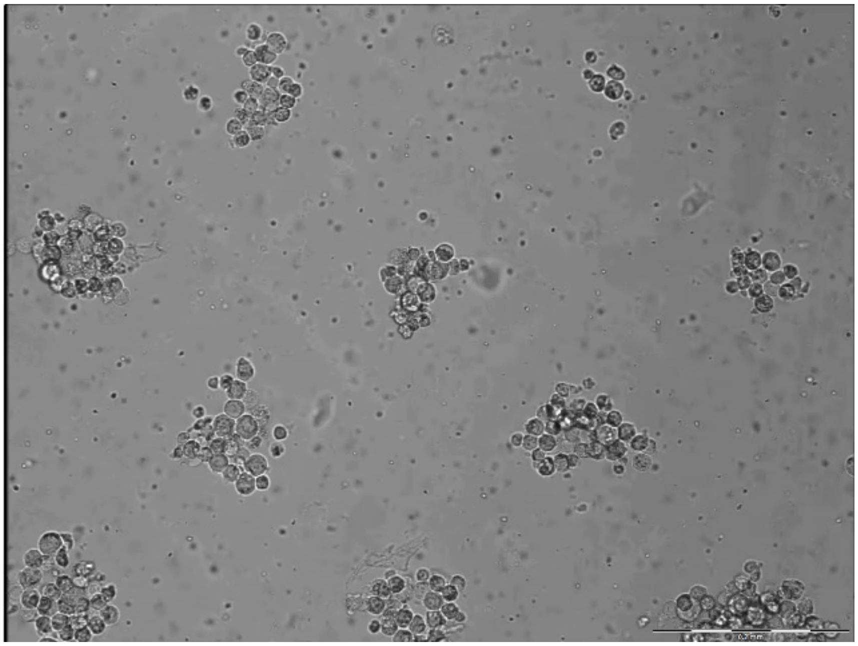

Supplementary Video 2: Spontaneous creation of 3D multicellular spheroids within hydrogel array from (B) E2-treated MCF7 cells.

See Supplementary Video 2

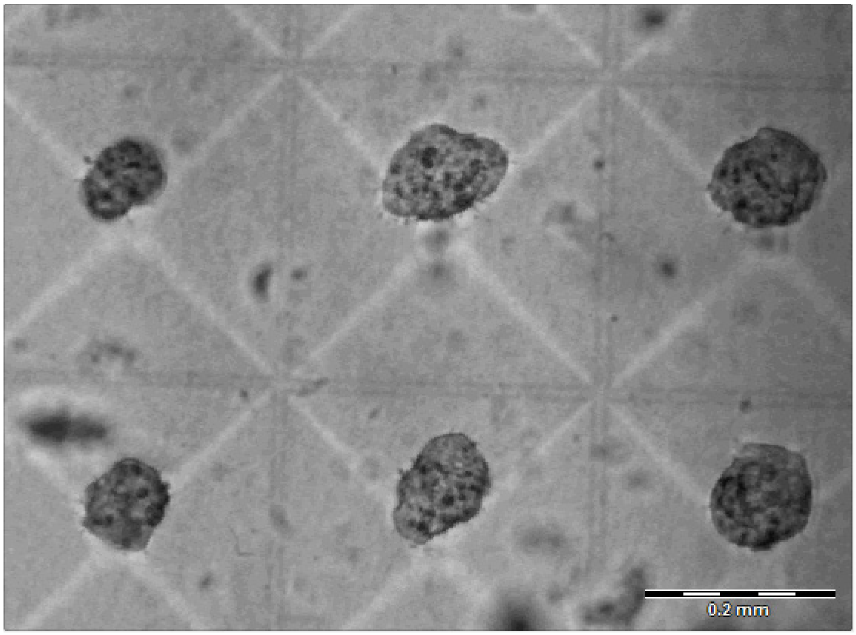

Supplementary Video 3: Effect of NO on 3D spheroid invasion within collagen matrix. (A) Non-treated spheroids without NO donor.

See Supplementary Video 3

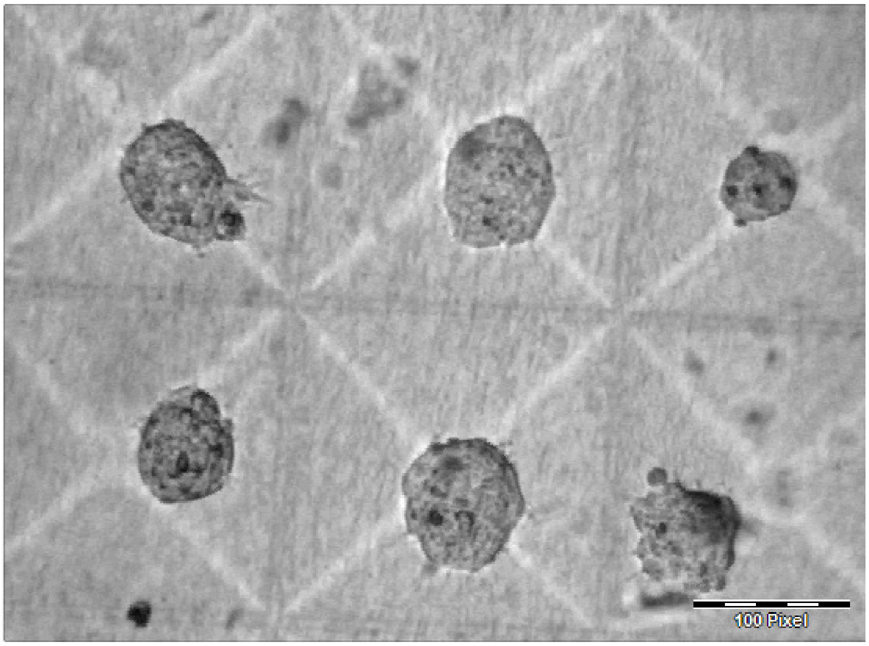

Supplementary Video 4: Effect of NO on 3D spheroid invasion within collagen matrix. (B) Non-treated spheroids in the presence NO donor.

See Supplementary Video 4

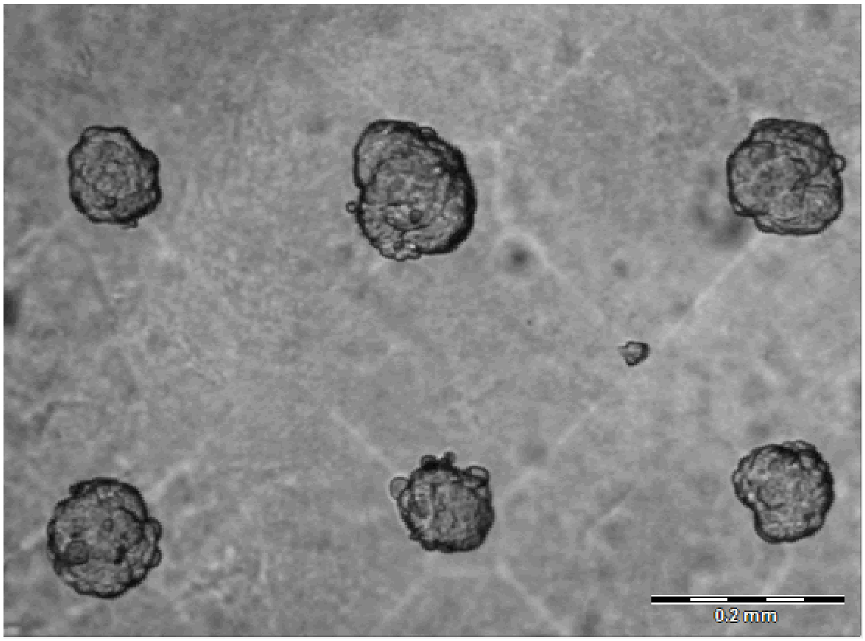

Supplementary Video 5:Effect of NO on 3D spheroid invasion within collagen matrix. (C) E2-treated spheroids without NO donor.

See Supplementary Video 5

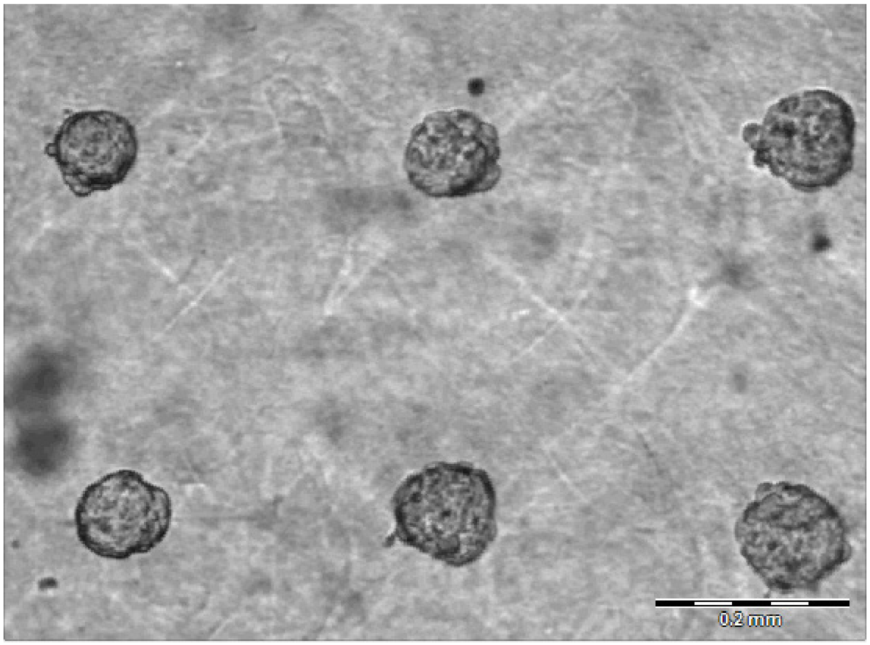

Supplementary Video 6: Effect of NO on 3D spheroid invasion within collagen matrix. (D) E2-treated spheroids in the presence of NO donor.

See Supplementary Video 6
