# Supplementary material for: Nitric oxide is cytoprotective to breast cancer spheroids vulnerable to estrogen-induced apoptosis
Source: Oncotarget. 2017 Oct 7;8(65):108890–911. doi: 10.18632/oncotarget.21610 (PMC5752490; doi:10.18632/oncotarget.21610)
Supplement: Supplementary file 2 [file oncotarget-08-108890-s002.docx]

**Nitric oxide is cytoprotective to breast cancer spheroids vulnerable to estrogen-induced apoptosis**

**Yana Shafran^1^*, Naomi Zurgil^1^*, Orit Ravid-Hermesh^1^, Maria Sobolev^1^, Elena Afrimzon^1^,
Yaron Hakuk^1^, Asher Shainberg^2^ and Mordechai Deutsch^1^**

**^1^The Biophysical Interdisciplinary Jerome Schottenstein Center for the Research and the Technology of the Cellome, Physics Department, Bar Ilan University, Ramat Gan 52900, Israel**

**^2^The Mina and Everard Goodman Faculty of Life Sciences, Bar Ilan University, Ramat Gan 52900, Israel**

*** Equal Contribution**

**Size and volume estimation of individual cells and spheroids.**

Individual MCF7 cells in suspension are spherical. For the estimation of size and volume, cells were seeded within HMC array and imaged by transmitted light. Due to the non-adherent nature of the hydrogel, breast cancer cells did not flatten and retained their globular shape. After defining ROIs for each cell, the area (Sc) of each ROI/cell was calculated utilizing Olympus Cell^P software, then cell radius was calculated and cell volume (Vc) was estimated as:


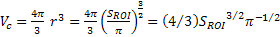

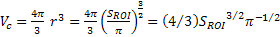


Most of the cell clusters which originated from both MCF7 phenotypes (E2-treated and non-treated) exhibited spherical arrangement 40h following seeding in HMC array (sphericity factor >0.6). Therefore size and volume of individual spheroids were assessed from their sectional area as described above. When possible, the average sectional area of 2-3 images of the same cell aggregates was calculated.

- For the estimation of the number of cells in each spheroid, it was first verified that spheroid volume was entirely packed with cells and there were no cavities or hollow spaces/areas. This was done using three methods:

1. Cell nuclei in intact spheroids were stained by Hoechst 33342 and then, imaged in several focal planes along the spheroid z axis by transmitted and fluorescent light. The number of nuclei in each plane was counted (See Supplementary Data, Supplementary Figure 1A-C).

2. Spheroids within the HMC array were fixed and inspected by histological examination (see Supplementary Data Supplementary Figure 1D).

3. 3D structures were generated within the array and imaged, followed by retrieval and dissociation into single cells.

All the above tests demonstrated that under the experimental conditions used here, MCF7 spheroids display compact multicellular structures that are filled with cells, at least during the first 4 days after generation. Moreover, there was a significant difference in the average cell size between the two spheroid phenotypes. Hence, estimation of the number of cells that comprise each spheroid was done through division of the spheroid volume by the corresponding average cell volume. The calculated number, size and volume of individual cells by image analysis correlated with the results obtained following spheroid dissociation followed by direct cell counting, and are also in agreement with other published works [1].


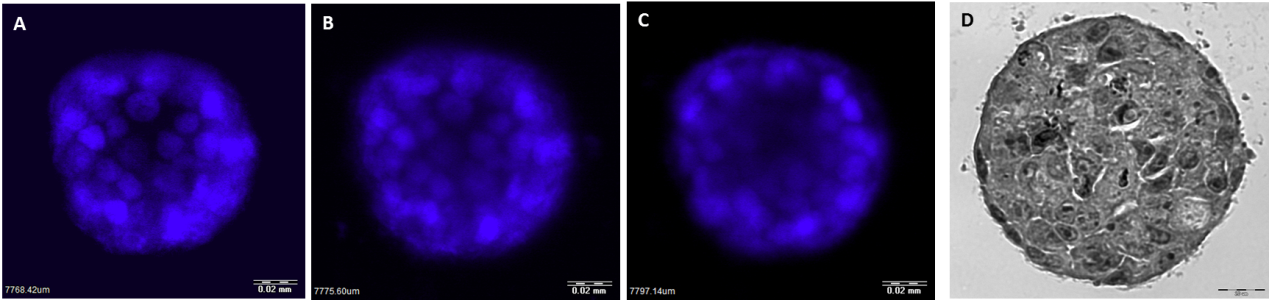


**Supplementary Figure 1: Calculating the number of nuclei/cells in BC spheroid.** Fluorescent images of a single spheroid following Hoechst 33342 staining at different focal planes **(A-C)**. Bright field image of BC spheroid following in-HMC array fixation and H&E staining **(D)**. Scale bar = 20μm

**Calculation of doubling time:**

Population doubling time of MCF7 cells grown as monolayer was calculated as:

Doubling time = {duration of culture (h) *log (2) / (log (Final concentration) - log (Initial concentration)}.

For spheroid population, estimation of the number of cells that comprise each individual spheroid was done as described above and doubling time was calculated as:

Spheroid doubling time = {duration of culture (h) *log (2) / (log (Final cell number) - log (Initial cell number)}.

**Reference**

1. Gunduz M, editor. Breast Cancer - Carcinogenesis, Cell Growth and Signalling Pathways InTech; 2011. Available from: http://www.intechopen.com/books/breast-cancer-carcinogenesis-cell-growth-and-signalling-pathways
