# Supplementary material for: Nitric oxide is cytoprotective to breast cancer spheroids vulnerable to estrogen-induced apoptosis
Source: Oncotarget. 2017 Oct 7;8(65):108890–911. doi: 10.18632/oncotarget.21610 (PMC5752490; doi:10.18632/oncotarget.21610)
Supplement: Supplementary file 3 [file oncotarget-08-108890-s003.docx]

**Nitric oxide is cytoprotective to breast cancer spheroids vulnerable to estrogen-induced apoptosis**

**Yana Shafran^1^*, Naomi Zurgil^1^*, Orit Ravid-Hermesh^1^, Maria Sobolev^1^, Elena Afrimzon^1^,
Yaron Hakuk^1^, Asher Shainberg^2^ and Mordechai Deutsch^1^**

**^1^The Biophysical Interdisciplinary Jerome Schottenstein Center for the Research and the Technology of the Cellome, Physics Department, Bar Ilan University, Ramat Gan 52900, Israel**

**^2^The Mina and Everard Goodman Faculty of Life Sciences, Bar Ilan University, Ramat Gan 52900, Israel**

*** Equal Contribution**

**TMRM staining and mitochondrial membrane potential analysis of 3D spheroids.**

TMRM, a fluorescent lipophilic cationic dye which accumulates within mitochondria in inverse proportion to Δψ_m,_ according to the Nernst equation, allows for comparative assessments of mitochondrial membrane potential among experimental conditions.

For quantitative analyses of the overall mitochondrial membrane potential of a cell cluster, two parameters were used: the TMRM ratio and TMRM CV. TMRM ratio ($TMRM Ratio=\frac{TMRM FI t=78h}{TMRM FI t=48h}$, where TMRM FI stands for the averaged FI of all the pixels within a spheroid area) reflects the change in mitochondrial membrane potential of a spheroid during the measurement period. TMRM CV, the spheroid TMRM coefficient of variation (CV) of pixel intensity ($TMRM CV=\frac{TMRM FI SD}{TMRM mean FI}$, where $TMRM FI SD$ stands for the standard deviation of spheroid TMRM FI, and $TMRM mean FI$ stands for the averaged spatial TMRM FI) represents the spatial distribution of TMRM FI within the spheroid area.

Live proliferating spheroids, exhibited an increase in mean TMRM FI signals during growth, resulting in TMRM ratio value higher than 1 (Supplementary Figure 2A). Although at each time point the correlation coefficient between individual spheroid size and the mean TMRM FI is low (Pearson correlation of 0.24, 0.05, 0.52 for 2, 3 and 4 days spheroid populations), the averaged increase in the spheroid size, accompanied higher TMRM FI signals (Supplementary Figure 2B).

Unlike the measurement of TMRM in individual cells, where the CV of the fluorescent signal represents the intracellular distribution of the probe, (e.g., mitochondria vs. cytoplasm) [1], TMRM CV parameter of a spheroid reproduces the spatial homogeneity of the fluorescent signal among all the cells that comprise the spheroid. Hence, low variation between individual cell FI values around the mean (reduced CV) reflects high spheroid metabolic activity, while a high rate of cell death or high proportion of low-metabolic-activity cells within the spheroid, results in high TMRM CV. This was confirmed by simultaneous staining of the same spheroids with the vital dye fluorescein diacetate (FDA). Kinetic measurements of the rate of fluorescein accumulation within the cells reflect both the esterase enzymatic activity and the integrity of the cell membrane. The rate of FDA hydrolysis was measured as described [2] and the linear slope of FI increase over time was calculated for each spheroid. Spheroid population (n=70) that exhibited the higher rates of fluorescein accumulation, displayed concomitantly elevated TMRM FI values and lower TMRM CV, while 3D structures of the same size (n=93) with low FDA hydrolysis rates, showed decreased mitochondrial membrane potential as reflected in the lower TMRM FI values and higher TMRM CV (see Supplementary Table 1).

**Supplementary Table 1: The rate of fluorescein accumulation and TMRM staining parameters in two BC spheroid populations having low and high metabolic activity**

|  | **FDA slope** | **TMRM FI [au]** | **TMRM SD** | **TMRM CV (%)** |
| --- | --- | --- | --- | --- |
| Low metabolic activity group (n=93) | 3.8±0.7 | 20.8±21 | 13.4±11.9 | 80±20 |
| High metabolic activity group (n=70) | 52.3±39 | 183.4±242.2 | 82.5±103.1 | 50±10 |

~~
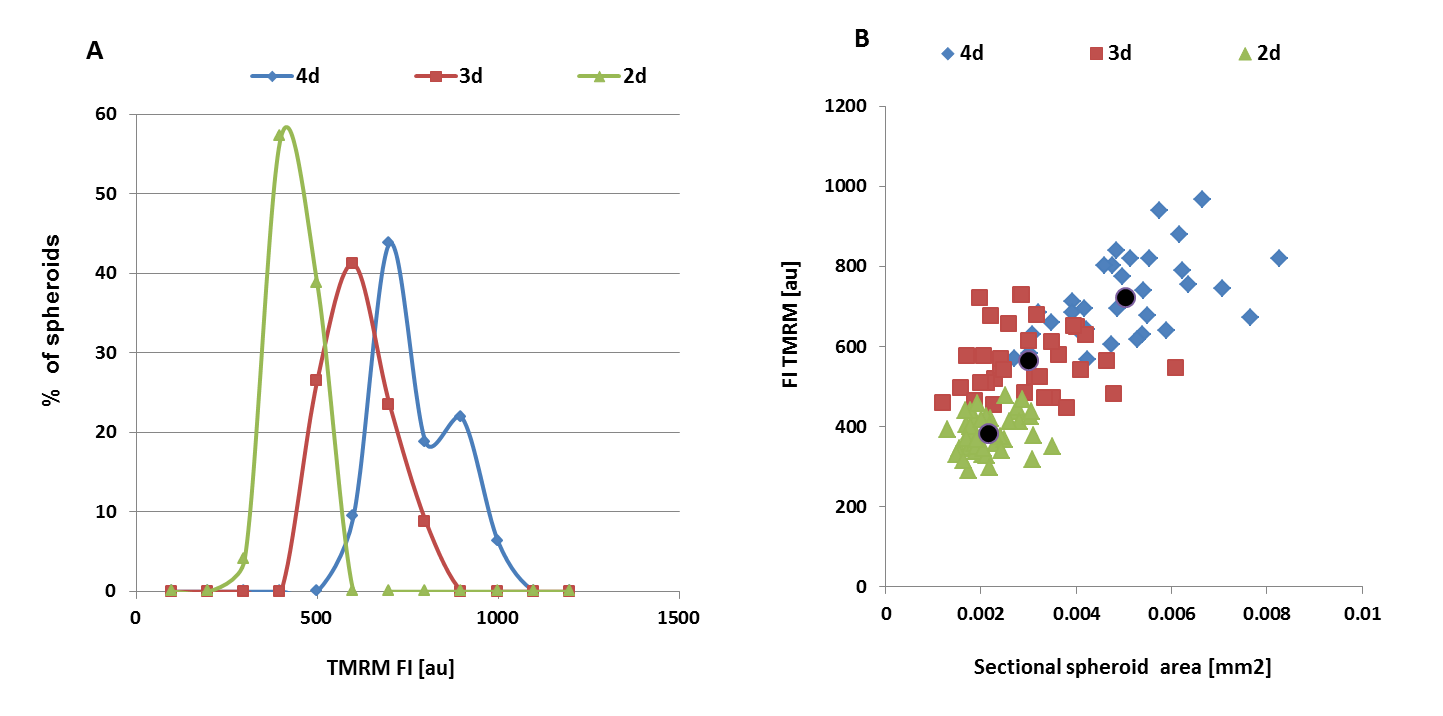
~~

**Supplementary Figure 2: MCF7 spheroid population was stained with TMRM at days 2, 3 and 4.** Distribution histograms of individual spheroid TMRM FI on each of the days is presented **(A)**. Correlation between spheroid size and TMRM FI signal during spheroid growth in culture **(B)**. Black dots represent mean signals of the spheroid populations.

**References**

1. Perry SW, Norman JP, Barbieri J, Brown EB, Gelbard HA. Mitochondrial membrane potential probes and the proton gradient: a practical usage guide. BioTechniques. 2011;50:98–115.

2. Afrimzon E, Zurgil N, Shafran Y, Ehrhart F, Namer Y, Moshkov S, Sobolev M, Deutsch A, Howitz S, Greuner M, Thaele M, Meiser I, Zimmermann H, Deutsch M. The individual-cell-based cryo-chip for the cryopreservation, manipulation and observation of spatially identifiable cells. II: functional activity of cryopreserved cells. BMC Cell Biol. 2010;11:83.
