# Supplementary material for: Nitric oxide is cytoprotective to breast cancer spheroids vulnerable to estrogen-induced apoptosis
Source: Oncotarget. 2017 Oct 7;8(65):108890–911. doi: 10.18632/oncotarget.21610 (PMC5752490; doi:10.18632/oncotarget.21610)
Supplement: Supplementary file 4 [file oncotarget-08-108890-s004.docx]

**Nitric oxide is cytoprotective to breast cancer spheroids vulnerable to estrogen-induced apoptosis**

**Yana Shafran^1^*, Naomi Zurgil^1^*, Orit Ravid-Hermesh^1^, Maria Sobolev^1^, Elena Afrimzon^1^,
Yaron Hakuk^1^, Asher Shainberg^2^ and Mordechai Deutsch^1^**

**^1^The Biophysical Interdisciplinary Jerome Schottenstein Center for the Research and the Technology of the Cellome, Physics Department, Bar Ilan University, Ramat Gan 52900, Israel**

**^2^The Mina and Everard Goodman Faculty of Life Sciences, Bar Ilan University, Ramat Gan 52900, Israel**

*** Equal Contribution**

**Estimation of intracellular NO concentration using DAF-2DA indicator.**

The compatibility of the imaging system and the HMC array-based imaging plate with quantitative NO fluorescence microscopic measurements was validated using a series of solutions that contained a fixed concentration of DAF-2 indicator and increasing concentration of NO-donor, as previously described [1]. For assessment of the endogenous levels of NO, live BC spheroids were loaded with DAF-2DA with and without the NOS inhibitor, L-NAME. Upon probe loading, the ester bonds of DAF-2DA are hydrolyzed by intracellular esterases, generating DAF-2, which accumulates within the cell. DAF-2 is non-fluorescent until oxidized by NO. An overlapping image of fluorescent and bright field images of non-treated BC spheroids loaded with DAF-2DA and settled within HMC array is presented in Supplementary Figure 3A.

The emitted fluorescence from a series of arrays containing spheroids that were pre-stained with 10 µM DAF-2DA and exposed to increasing concentrations of DETA/NO for 0.5h at 37˚C in a CO_2_ humidified incubator was used for constructing the calibration curve. The FI values of individual spheroids were extracted using image analysis, and the population averaged intracellular FI was calculated. Under these conditions, (objective x20), the calibration curve obtained for intra-cellular NO levels measured, showed good linearity within the range of NO-donor concentrations used, (R2=0.989, Fig SD3B) and the detection limit of in-HMC fluorescence based NO measurement was about 40 nM. However, given the well-reported difficulties in the absolute measurement of NO concentration, and the fact that NO modulates on its own synthesis, these fluorescence measurements can only provide information on relative levels.


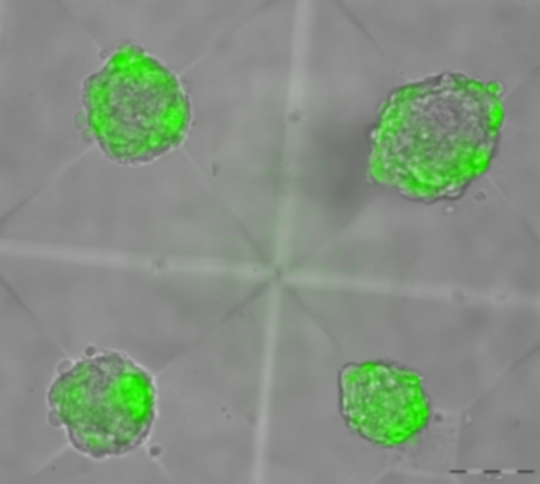


**A**


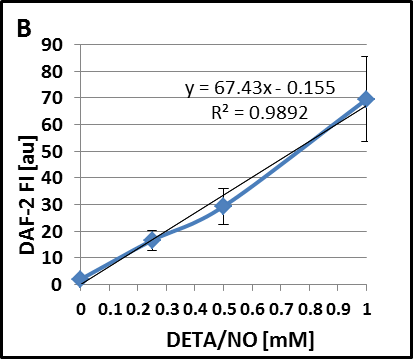


**Supplementary Figure 3: (A)** An overlapping image of fluorescent and bright field images of representative non-treated BC spheroids stained with DAF-2DA and settled within HMC array. Bar represents 100µM. **(B)** Average DAF-2 FI values of BC spheroid populations in response to increased concentration of NO donor. Each dot represents an average ±SD of at least 100 individual spheroids.

Using the calibration curve, the population-averaged FI signals (n=200 spheroids) were estimated to be equivalent to NO concentrations of 80 nM and 140 nM in non-treated and E2-exposed spheroid populations, respectively. In the presence of the NOS inhibitor L-NAME, an average decrease of about 30% in mean FI values was evident in hormone-deprived spheroid populations, while only a small change (5%) in DAF-2 FI was observed in hormone-exposed spheroids. These results indicate that about a third of the FI signal originates from de novo enzymatic production of NO in non-treated cell clusters, but in E2-treated spheroids which undergo extensive apoptosis, most of the DAF-2 fluorescent signal is derived from non-enzymatic source. Taken together, these data indicate that under the experimental conditions used here, the estimated relative NO level in E2-exposed spheroids is about double the level in hormone-deprived BC spheroids. Furthermore, the proportion of NOS-based NO concentration is higher in non-treated spheroids.

**Reference**

1. Shafran Y, Zurgil N, Afrimzon E, Tauber Y, Sobolev M, Shainberg A, Deutsch M. Correlative analyses of nitric oxide generation rates and nitric oxide synthase levels in individual cells using a modular cell-retaining device. Anal Chem. 2012;84:7315–22.
