# Supplementary material for: Nitric oxide is cytoprotective to breast cancer spheroids vulnerable to estrogen-induced apoptosis
Source: Oncotarget. 2017 Oct 7;8(65):108890–911. doi: 10.18632/oncotarget.21610 (PMC5752490; doi:10.18632/oncotarget.21610)
Supplement: Supplementary file 5 [file oncotarget-08-108890-s005.docx]

**Nitric oxide is cytoprotective to breast cancer spheroids vulnerable to estrogen-induced apoptosis**

**Yana Shafran^1^*, Naomi Zurgil^1^*, Orit Ravid-Hermesh^1^, Maria Sobolev^1^, Elena Afrimzon^1^,
Yaron Hakuk^1^, Asher Shainberg^2^ and Mordechai Deutsch^1^**

**^1^The Biophysical Interdisciplinary Jerome Schottenstein Center for the Research and the Technology of the Cellome, Physics Department, Bar Ilan University, Ramat Gan 52900, Israel**

**^2^The Mina and Everard Goodman Faculty of Life Sciences, Bar Ilan University, Ramat Gan 52900, Israel**

*** Equal Contribution**

**Design and fabrication of hydrogel array and imaging device.**

A hydrogel microchamber (HMC) array based imaging plate was developed and fabricated by embossing the hydrogel array at the inner surface of the glass bottom (170 μm height) of commercial 6- or 24-well imaging plates as described [1]. In brief, for production of PDMS stamps with a negative microchamber (MC) array, an array of square bottom pyramid shaped MCs was made by selective DRIE etching of a silicon oxide wafer patterned using conventional photolithography (GeSiM mBH, Germany) and coated with teflon to reduce peeling tension in the PDMS.

For production of hydrogel array, a small drop of warm LMA was symmetrically dripped on the surface of the plate’s glass bottom, and the pre-heated PDMS stamp was then gently placed over the LMA. The system was incubated at RT for 5-7min for pre-gelling and pre-cooling, followed by 5-10 min incubation at 4ºC for LMA gelation. At the culmination of gelation process, the PDMS stamp was peeled off, leaving agarose gel patterned with MCs. The imaging plate, consisting of optical bottom patterned with HMC array was UV sterilized and stored at 4ºC in humidified atmosphere up to one month.

**Cell loading and spheroid generation.**

For loading into MC array, cells were collected by trypsinization and manual scraping, washed and suspended at appropriate concentrations in fresh complete medium.

A cell suspension (30-50 µL, 70-120×10^3^ cells per mL, in medium) was gently loaded on top of the HMC array and cells were allowed to settle by gravity for 15min. Next, 4-8 aliquots of 250 µL fresh medium (total 1-2 mL) for the 24- or 6-well imaging plate, respectively, were gently added to the rim of the macro-well plastic bottom beside the hydrogel array. For medium exchange, 900-1900 μL medium were removed from the macro-chamber and exchanged. In experiments where multiple medium exchange and treatments were performed (e.g. multiple staining, cell fixation and immunofluorescence staining), in order to keep the non-tethered spheroids in their original positions, the following procedure was performed: after 24h of spheroid generation, medium was removed and spheroids were covered by 0.5% warm LMA (50-100 µL), incubated at RT for 5-7min and at 4ºC for 2min. Then, 1-2mL of warm medium was added. This procedure does not affect any of the tested parameters such as spheroid growth, fluorescent staining and immunofluorescence staining

**Imaging system and operating software.**

Images were acquired using a motorized Olympus inverted IX81 microscope (Olympus Corporation, Tokyo, Japan). The microscope is equipped with a sub-micron Marzhauser Wetzlar motorized stage type SCAN-IM, with an Lstep controller (Marzhauser Wetzlar GmbH, Wetzlar, Germany) and a filter wheel including a fluorescence cube (excitation filters, dichroic mirrors and emission filters, respectively) for FITC, 470-490nm, 505nm long pass and 510-530nm, and for Hoechst 33342, 355-405nm, 410nm long pass and 420-450nm, for TMRM, 530-560nm, 570nm and 590nm long pass. All filters were obtained from Chroma Technology Corporation (Brattleboro, VT, USA).

Objectives of ×4/×10/×20 were used for HMC array image acquisition. The complete microscope system was enclosed with an incubator which provided a temperature of 37°C and humidified atmosphere containing 5% CO2 (Life Imaging Services, Switzerland), allowing monitoring over long periods.

A cooled, highly sensitive 14-bit, ORCA II C4742-98 camera (Hamamatsu Photonics, Hamamatsu, Japan) was used for imaging. The Olympus Cell^P, (Olympus Corporation, Tokyo, Japan), ImageJ or MATLAB software were used for image analysis.

**Reference**

1. Afrimzon E, Botchkina G, Zurgil N, Shafran Y, Sobolev M, Moshkov S, Ravid-Hermesh O, Ojima I, Deutsch M. Hydrogel microstructure live-cell array for multiplexed analyses of cancer stem cells, tumor heterogeneity and differential drug response at single-element resolution. Lab Chip. 2016;16:1047–62.
